# Update on *Streptococcus suis* Research and Prevention in the Era of Antimicrobial Restriction: 4th International Workshop on *S. suis* †

**DOI:** 10.3390/pathogens9050374

**Published:** 2020-05-14

**Authors:** Mariela Segura, Virginia Aragon, Susan L. Brockmeier, Connie Gebhart, Astrid de Greeff, Anusak Kerdsin, Mark A O’Dea, Masatoshi Okura, Mariette Saléry, Constance Schultsz, Peter Valentin-Weigand, Lucy A. Weinert, Jerry M. Wells, Marcelo Gottschalk

**Affiliations:** 1 Research Group on Infectious Diseases in Production Animals and Swine and Poultry Infectious Diseases Research Centre, Faculty of Veterinary Medicine, University of Montreal, St-Hyacinthe, QC J2S 2M2, Canada; 2 IRTA, Centre de Recerca en Sanitat Animal (CReSA, IRTA-UAB), Campus de la Universitat Autònoma de Barcelona, 08193 Bellaterra, Spain; virginia.aragon@irta.es; 3 USDA, ARS, National Animal Disease Center, Ames, IA 50010, USA; susan.brockmeier@usda.gov; 4 College of Veterinary Medicine, University of Minnesota, St. Paul, MN 55108, USA; gebha001@umn.edu; 5 Wageningen Bioveterinary Research, 8221 RA Lelystad, The Netherlands; astrid.degreeff@wur.nl; 6 Faculty of Public Health, Kasetsart University Chalermphrakiat Sakon Nakhon Province Campus, Sakon Nakhon 47000, Thailand; Anusak.ke@ku.th; 7 Antimicrobial Resistance and Infectious Disease Laboratory, School of Veterinary and Life Sciences, Murdoch University, Perth, Western Australia 6150, Australia; m.odea@murdoch.edu.au; 8 Division of Bacterial and Parasitic Diseases, National Institute of Animal Health, National Agriculture and Food Research Organization, Tsukuba, Ibaraki 305-0856, Japan; mokura@affrc.go.jp; 9 French Agency for Veterinary Medicinal Products-French Agency for food, Environmental and Occupational Health Safety (Anses-ANMV), 35302 Fougères, France; mariette.salery@anses.fr; 10 Department of Global Health-Amsterdam Institute for Global Health and Development and Department of Medical Microbiology, Amsterdam University Medical Centers, University of Amsterdam, 1105 BP Amsterdam, The Netherlands; c.schultsz@aighd.org; 11 Institute for Microbiology, University of Veterinary Medicine, 30173 Hannover, Germany; Peter.Valentin@tiho-hannover.de; 12 Department of Veterinary Medicine, University of Cambridge, Cambridge CB3 0ES, UK; lw461@cam.ac.uk; 13 Host-Microbe Interactomics Group, Department Animal Sciences, Wageningen University and Research, 6709 PG Wageningen, The Netherlands; jerry.wells@wur.nl; 14 Department of Veterinary Medicine, University of Cambridge, Cambridge CB3 0ES, UK

**Keywords:** *Streptococcus suis*, swine, zoonosis, epidemiology, genomics, diagnosis, antimicrobials, vaccines, public health, vaccine policies

## Abstract

*Streptococcus suis* is a swine pathogen and a zoonotic agent afflicting people in close contact with infected pigs or pork meat. Sporadic cases of human infections have been reported worldwide. In addition, *S. suis* outbreaks emerged in Asia, making this bacterium a primary health concern in this part of the globe. In pigs, *S. suis* disease results in decreased performance and increased mortality, which have a significant economic impact on swine production worldwide. Facing the new regulations in preventive use of antimicrobials in livestock and lack of effective vaccines, control of *S. suis* infections is worrisome. Increasing and sharing of knowledge on this pathogen is of utmost importance. As such, the pathogenesis and epidemiology of the infection, antimicrobial resistance, progress on diagnosis, prevention, and control were among the topics discussed during the 4th International Workshop on *Streptococcus suis* (held in Montreal, Canada, June 2019). This review gathers together recent findings on this important pathogen from lectures performed by lead researchers from several countries including Australia, Canada, France, Germany, Japan, Spain, Thailand, The Netherlands, UK, and USA. Finally, policies and recommendations for the manufacture, quality control, and use of inactivated autogenous vaccines are addressed to advance this important field in veterinary medicine.

## 1. Introduction

**Figure 1:**
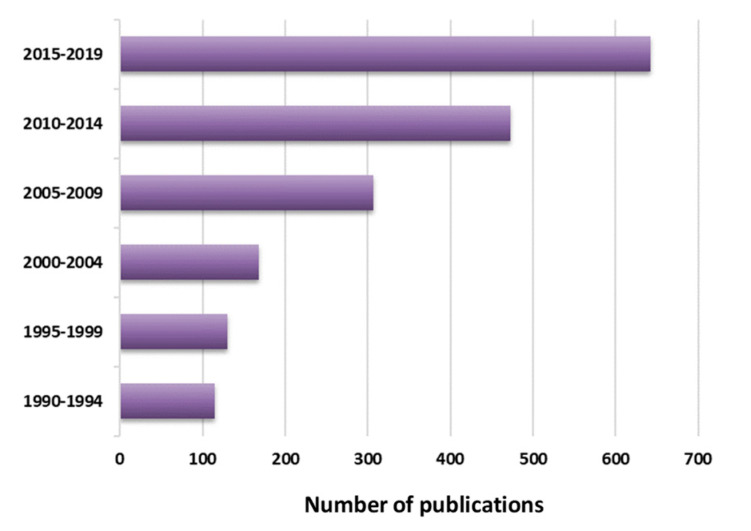
Progression in the number publications on *S. suis* per 5-year periods since 1990. Source: PubMed (https://www.ncbi.nlm.nih.gov/pubmed).

*Streptococcus suis* is considered one of the most important bacterial swine pathogens leading to important economic losses to the porcine industry worldwide. *S. suis* has been reported globally in both traditional and intensive swine operations [1]. Control is based on an alarming overuse of antimicrobials, leading to a dramatic increase of the risk related to antimicrobial resistance. It is also an agent of disease in humans and considered in most OECD (Organisation for Economic Co-operation and Development) countries as an occupational disease affecting mostly swine industry workers. In Asia, this pathogen affects the general population and represents a significant public health concern [2]. After a deadly 2005 Chinese human outbreak, research teams worldwide turned their attention to *S. suis* with an explosion of published articles (Figure 1). 

In 2013, the 1st International Workshop on *S. suis* was organized with the aim to increase international collaborations. Since then, the upsurge of studies certainly contributed to our understanding of bacterial–host interactions. However, the use of different research models resulted in misconceptions and complicated diagnostics and vaccine development [3,4]. Eight years after the 1st workshop and after two other workshops (2014 and 2016), the 4th International Workshop on *S. suis* was organized to strengthen scientific knowledge and provide, through international cooperation, relevant scientific information, and advice that will have a direct influence on the decisions made by the swine industry. 

The diagnosis and epidemiology of the infection in humans and pigs; different aspects of the pathogenesis of the disease; antimicrobial resistance, prevention and control; and finally autogenous vaccine policy were addressed during the meeting and are further discussed below.

## 2. Diagnosis and Epidemiology of the *S. suis* Infection in Humans and Pigs

*S. suis* is an encapsulated pathogen, and the capsular polysaccharide (CPS) antigen is the basis of *S. suis* classification into serotypes [1]. Originally, 35 serotypes were identified. However, phylogenetic and/or sequence analyses showed that the reference strains of serotypes 20, 22, 26, 32, 33, and 34 should be taxonomically removed from the *S. suis* species. Serotypes 32 and 34 were reclassified as *Streptococcus orisratti*. Serotypes 20, 22, and 26 were proposed as *Streptococcus parasuis*, while serotype 33 was classified as *Streptococcus ruminantium* [5,6,7]. Nevertheless, more extensive studies using a higher number of *S. suis* and *S. suis*-like strains will provide additional insights into the classification of these strains and make the species boundaries clearer [6]. In fact, all 35 serotypes of *S. suis* (or *S. suis*-like strains) (with the exception of serotype 33) are isolated from diseased pigs [1], and from the clinical point of view, many diagnostic laboratories still identify the 35 serotypes. The tools for molecular epidemiology of *S. suis* have been recently reviewed in Reference [7].

**Figure 2:**
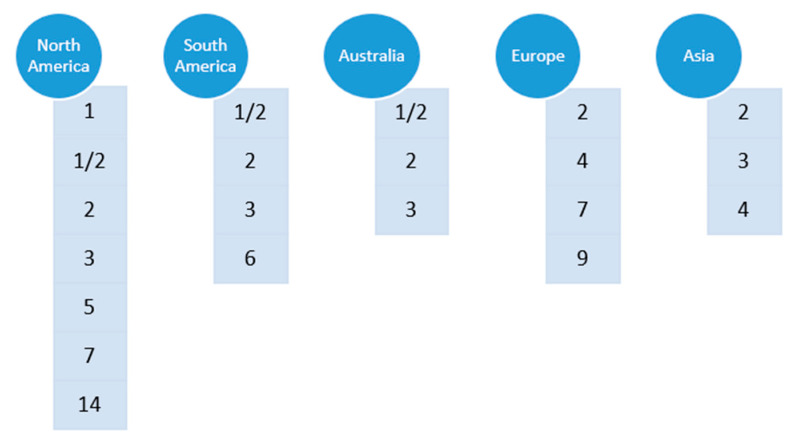
Schematic representation of worldwide distribution of major *S. suis* serotypes involved in swine clinical cases: The listed order of serotypes does not reflect the relative frequencies of each serotype, as they might vary from one country to another.

The worldwide distribution of major *S. suis* serotypes involved in swine clinical cases is schematically represented in Figure 2. Among those reported, serotype 2 is considered the most common cause of infections in piglets worldwide and a major zoonotic agent [2]. Nevertheless, other serotypes are increasing in importance in different countries, as is the case of serotype 9, particularly in some countries of Western Europe. By means of novel animal models and diagnostic tools, de Greeff et al. (Appendix A) epidemiologically determined the population genetics of *S. suis* serotype 9 in The Netherlands [8]. Obtained data using comparative genome hybridization and whole genome sequencing suggest that clinical serotype 9 swine isolates are genetically very similar whereas serotype 9 isolates carried by healthy pigs are more heterogeneous. A few carriage isolates clustered together with clinical isolates; these carriage isolates probably reflect clinical isolates that are not causing any clinical outbreaks on the farms but do have virulent potential. By infecting Caesarean Derived Colostrum Deprived (CDCD) piglets intravenously with a high dose of bacteria, it was shown that, within *S. suis* serotype 9, the virulence of clinical and tonsillar carriage isolates differs significantly (Appendix A). Interestingly, a recent study indicated that *S. suis* isolates associated with disease in pigs comprise predominantly of serotypes 2, 3, and 1/2 (Appendix B), which is consistent with reports from other pig producing countries [2].

In Figure 2, it can also be appreciated that the epidemiological situation in North America is different from other countries. In this part of the globe, multiple serotypes are found in swine clinical cases [2]. Indeed, recent works confirmed that a variety of *S. suis* serotypes can be found in commercial swine production systems in USA and Canada, with serotypes 1/2, 7, 2, 1, 3, and 5 commonly isolated from systemic infection sites, the frequencies depending on the study [9,10]. 

**Figure 3:**
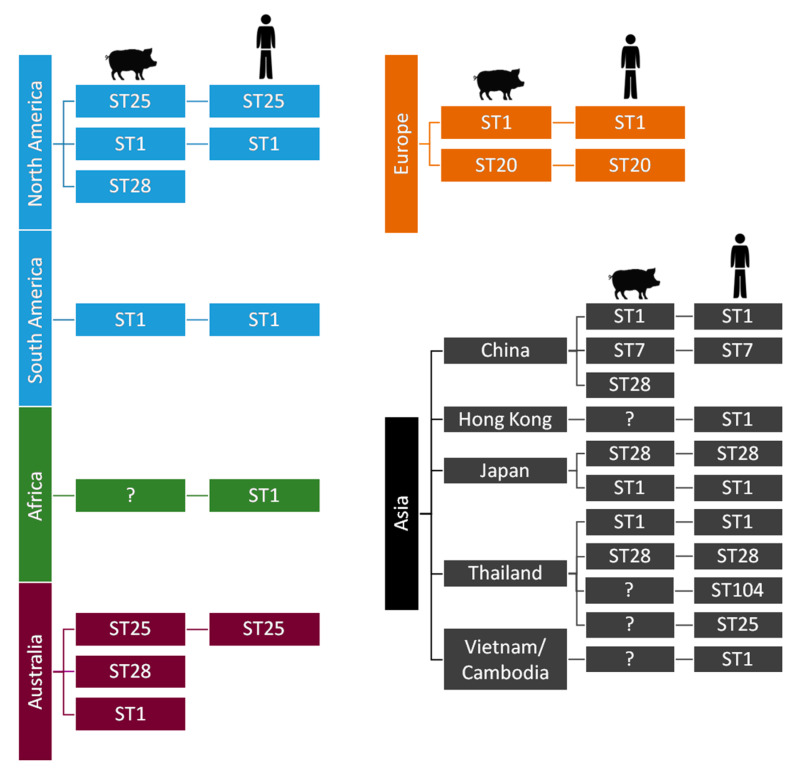
Most important sequence types (STs) of *Streptococcus suis* serotype 2 as determined by multilocus sequence typing (MLST): ST1 serotype 2 strains are mostly associated with disease in both pigs (where data are available) and humans in Europe, Asia, Africa, and South America. The situation is different in North America, where fewer clinical ST1 cases of infection in pigs and only one human ST1 case has been described. ST7, a single locus variant of ST1, is endemic to mainland China. Interestingly, Japan and Thailand are the only countries reporting ST28 human cases [2]. “?” means no data available from swine clinical cases.

Besides this classification based on CPS antigen/gene locus, *S. suis* is genetically differentiated into sequence types (ST) by multilocus sequence typing (MLST) [7]. The distribution of most important STs within serotype 2 is represented in Figure 3. 

It is important to note that epidemiological data is missing for several countries; for example, information relating to *S. suis* in the Australian pig herd is limited [11,12]. To fill this gap, O’Dea et al. (Appendix B) analysed and characterised a significant number of *S. suis* isolates from diseased pigs obtained across multiple production sites over a seven year period [13]. The most prominent MLSTs were, in decreasing order, ST27, ST25, ST28, ST483, and ST1. Analysis of serotype and MLST combinations showed a high proportion of isolates as serotype 2 ST25, serotype 3 ST27, and serotype 2 ST28. A deeper phylogenetic comparison of the *S. suis* isolates from Australian pigs against a global collection of *S. suis* strains showed that Australian clones of *S. suis* associated with clinical disease in pigs have maintained a stable core genome, mirroring the international seed-stock from which they were derived (mainly from the UK and North America). While Australian serotype 2 ST25 isolates could be distinguished from those of USA and Canada, only small single nucleotide polymorphism (SNP) differences are notable across the core genome. Despite the limited number, the characterisation of serotype 1/2 ST1 clones is significant, a finding also recently observed in North America. Indeed, Gebhart et al. (Appendix C) characterised the diversity of a contemporary collection of *S. suis* isolates across North America (mainly in USA) by serotyping and MLST to address the limited information on current *S. suis* strains circulating within this country [10]. The predominant ST was ST28, followed by ST94, ST1, and ST108 among multiple STs identified, illustrating the high diversity among *S. suis* isolates. Importantly, this study revealed the predominance of serotype 1/2 ST28 from clinically affected pigs in USA. In addition, the study also reported ST1 strains, containing the three classical virulence-associated genes (*epf*, *sly*, and *mrp*), which are considered highly virulent and potentially zoonotic, an unexpected finding based on previous epidemiological data [2]. Epidemiological surveillance of these strains in North America is recommended [10]. 

*S. suis* is a zoonotic disease of increasing awareness. Sporadic cases of human infection have been described worldwide since 1968; however, multiple outbreaks in some Asian countries highlight the public health importance of this infection [2,14,15,16] (Appendix D). Small skin wounds are the main route of entry in humans in Western countries, although in some cases no wound is evidenced [1]. In some Asian countries, such as Vietnam and Thailand, human *S. suis* is considered among the most frequent causes of bacterial meningitis in adults [17,18]. Cultural differences between Asian and Western countries likely impact the epidemiology of *S. suis* including lifestyle; common use of backyard production systems; close contact of humans with pigs; and, in countries such as Vietnam, Laos and Thailand, the common practice of consuming raw pork products [19,20,21]. It has been clearly demonstrated that the oral route of infection is the principal cause of human infection in these countries [1,20]. As aforementioned, serotype 2 is the main zoonotic serotype; however, serotype 14 has also been isolated from humans many times in Thailand and UK and uncommonly in France, Australia, and Canada [1,22]. After serotype 14, serotype 5 is the third serotype most commonly found from humans [23]. Sporadic cases due to other serotypes have also been reported [2]; among them, serotype 9, one of the most important serotype recovered from diseased pigs in Europe, has been recently shown to have zoonotic potential [24]. 

Within serotype 2, STs involved in human cases are represented in Figure 3 and parallel those found in swine clinical cases, highlighting their epidemiological link. Kerdsin et al. (Appendix D) have been extensively studying the epidemiological situation in Thailand [14,22]. To date, four outbreaks of *S. suis* infections in humans have been recorded in that country, with case fatality rates varying from 6.5% to 16.1%. The food safety campaign implementation in Phayao province during 2011–2013 showed a marked decrease of the disease incidence proportion and stresses the need for health policies to reduce the burden of this infection [20]. Microbiological characterisation of *S. suis* in Thailand showed that serotype 2 is the main serotype for human infections, followed by serotypes 14, 24, 5, 4, 9, and 31. Of note, ST1 and ST104 (serotype 2) are predominant in Thai human infections. Interestingly, the latter is almost exclusively found in that country. On the other hand, ST105 is the main ST among serotype 14 isolates from human cases. Kerdsin et al. also revealed that Thai serotype 9 isolates are of ST16 (Appendix D) [24], also commonly affecting pigs in some European countries [2,25]. Schultsz et al. (Appendix E) have been studying the emergence of zoonotic *S. suis* infections in The Netherlands. Zoonotic *S. suis* serotype 2 strains of ST20, isolated from human patients, were shown to be highly genetically related to serotype 9 strains of ST16 [25]. It has been shown that the CPS is a major virulence factor (see Section 3) and that the *cps* gene locus can be exchanged between *S. suis* of different serotypes. Interestingly, a study from The Netherlands [26] showed that such CPS switch may lead to an increase in zoonotic potential. In addition to *cps* locus acquisition, loss or acquisition of other genes or loci may contribute to changes in zoonotic potential. For example, the *S. suis* ST20 strains (typically found in The Netherlands) acquired not only the serotype 2 *cps* locus but also a type 1 Restriction-Modification (R-M) system. Schultsz et al. (Appendix E) suggested that *S. suis* ST20 strains may have acquired the ability to adapt to specific niches through the acquisition of a R-M system that can regulate expression of CPS and/or other (surface exposed) molecules. 

Besides serotype 2 ST1 strains, which present high zoonotic potential worldwide, serotype 2 ST7 is only endemic to China, and it was responsible for human outbreaks in 1998 and 2005 in this country [16]. In a recent study addressing the evolution of ST7 strains in China, it was shown that, of 38 sporadic ST7 *S. suis* strains, which mostly caused sepsis, serotype 14 was the most frequent, followed by serotype 2. Compared to the genome of the epidemic strain (serotype 2, ST7), the major differences in the genomes of sporadic ST7 strains were the absence of the 89 kb pathogenicity island specific to the epidemic strain and insertion of mobile elements that play a significant role in the horizontal transfer of antimicrobial resistance genes [27]. This is the first study addressing the evolution of the ST7 strains and reporting serotype 14 ST7 isolates, highlighting the need to increase the surveillance of this human life-threatening lineage of *S. suis*.

In addition to the “classical” described serotypes, a novel variant (serotype Chz) and strains carrying 26 novel capsular polysaccharide loci (NCL1-26) have been identified recently [28,29,30,31,32]. These findings expand the views of the genetic diversity of *S. suis cps* loci. Novel *cps* loci are continually being found, and their discovery should eventually reduce the number of untypeable strains recovered from diseased animals. Nevertheless, their virulence potential and the role of these NCLs in the pathogenesis of the disease remain to be evaluated.

## 3. Virulence Factors and Pathogenesis of the Infection

In pigs, *S. suis* usually colonises the upper respiratory tract, in particular the pharyngeal and palatine tonsils, but alimentary and genital tracts can also be colonisation sites. In fact, piglets are first colonised by *S. suis* from birth as soon as they pass through the birth canal, since *S. suis* is found in the sow vagina. In humans, *S. suis* nasopharyngeal colonisation has been reported in people working in close contact with infected animals, such as butchers and abattoir workers [1,19,33]. The bacteria may also colonise the gastrointestinal tract in people consuming fresh/raw contaminated pork meat [19]. Consequently, the mucosal barrier is the first line of defence of the host against this pathogen. In swine, colonisation of the upper respiratory tract by *S. suis* may lead to an asymptomatic carriage but is also considered the first step for the development of an invasive disease, particularly in the context of coinfections with porcine respiratory viruses or polymicrobial infections [34,35,36,37,38,39,40,41,42]. 

Indeed, *S. suis* belongs to the “porcine respiratory disease complex”, a term used to describe a common multifactorial respiratory disease in swine that occurs as a result of polymicrobial infections, environmental stressors, and host factors such age and immunological status. Brockmeier et al. (Appendix F) have been analysing the complexity of these polymicrobial infections, which can be the result of multiple viruses, bacteria, or a combination of viruses and bacteria infecting the pig at the same time or in close succession. For example, the majority of cases where porcine reproductive and respiratory syndrome virus (PRRSV) or swine influenza virus (SwIV) was determined to be the primary aetiology, also presented with secondary bacterial pneumonia caused by *S. suis*, among other bacterial species. Experimental data suggest that coinfections with viruses, such as PRRSV, SwIV, porcine circovirus, and porcine respiratory coronavirus (PRCV), generally result in a more severe clinical disease outcome. There are several mechanisms that might contribute to increased susceptibility to secondary infection and the enhanced disease that often occurs with coinfections, including disruption of the epithelial barrier or alteration of the innate or adaptive immune responses by the primary pathogen, direct interactions between or among the pathogens such as the formation of multispecies biofilms, disturbances to the ecological niche such as the upper respiratory microbiota, or enhanced transmission. Brockmeier et al. (Appendix F) and others [39,43,44,45] have shown that coinfection with PRRSV, SwIV or PRCV and bacteria such as *Bordetella bronchiseptica*, *S. suis* or *Glaesserella (Haemophilus) parasuis* results in a greater incidence of disease, greater percentage of lungs affected, more severe lesions, and slower resolution of such lesions than occurs with infection with a single pathogen alone and that this is often correlated with an amplified local pro-inflammatory cytokine response.

In the absence of appropriate models of polymicrobial infections typically seen in swine production facilities, mechanistic understanding of the underlying complex molecular and cellular interactions remains limited. The transition between *S. suis* colonisation of the mucosal barriers and systemic infection is only partially understood. In the studies performed by Wells et al. [46], a hypothetical model to explain systemic infection of piglets with *S. suis* based on the identification of *S. suis* in the tonsillar lymphoid tissue and presence of large numbers of CD169+ macrophages was proposed. In this model, CD169+ macrophages might act as reservoirs for replication of virulent strains of *S. suis*, whereby environmental and physical stressors that reduce competition from the tonsil microbiota lead to increased *S. suis* in the tonsil and escape into the bloodstream through the many small blood vessels permeating the lymphoid tissue. Such a model would also be compatible with the finding that serotype 2 strains interacting with the CD169 Siglec 1 receptor through their sialylated CPS are more commonly associated with invasive disease. Wells et al. also proposed that subversion of the innate functions of CD169+ macrophages by coinfecting viruses such as PRRSV or SwIV may also contribute to the survival of *S. suis* and spread to the bloodstream. In this context, the potential mechanisms used by *S. suis* serotypes without sialic acid in their CPSs are largely unknown. It is also possible that survival and or replication capacity of *S. suis* in this particular subset of macrophages is a characteristic associated with disease-causing strains. 

In this regard, research performed by Valentin-Weigand et al. (Appendix G) has significantly contributed to our understanding of viral–bacterial interactions during airway coinfections. One important suggested virulence factor is the *S. suis* cholesterol-dependent cytotoxin, named suilysin, produced by many virulent strains. This toxin has multiple actions towards a variety of cells (reviewed in Reference [47]), including attachment of *S. suis* to the surface of the respiratory epithelium and/or direct lysis of epithelial cells [48,49], probably facilitating bacterial breach of this mucosal barrier. However, suilysin-negative clinical isolates are also found frequently in swine populations worldwide and especially in North America [10,50]. Valentin-Weigand et al. found that, during secondary bacterial infection, suilysin contributes to the damage of well-differentiated respiratory epithelial cells in the early stage of infection, whereas cytotoxic effects induced by SwIV became prominent at later stages of infection. Besides suilysin, it has been shown that a prior infection by SwIV enhances the adherence to and colonisation of porcine airway epithelial cells by *S. suis* in a sialic-acid dependent manner and facilitates invasion in a suilysin-independent fashion [51,52,53]. These findings might explain why suilysin-negative strains can also cause clinical infections (Appendix G). As aforementioned, the potential mechanisms used by *S. suis* serotypes without sialic acid in their CPSs remain to be discovered.

In humans, the intestinal route of infection seems to be an important port of entry after consumption of fresh/raw contaminated pork meat in some Asian countries. In pigs, this route of infection has also been investigated by direct inoculation in the intestine or oral capsule-mediated delivery of virulent strains of *S. suis* into the small intestine of piglets [54,55]. In view of the inoculum size, stress-inducing conditions, and the small proportion of challenged animals which developed invasive disease in those studies, translocation from the intestine appears to be a possible but not a very efficient route of infection. The conditions leading to sufficient passage of *S. suis* through the stomach are still unclear and might differ in neonatal, suckling, or weaning periods. More studies are required on oro-gastrointestinal *S. suis* infections in piglets to confirm the relevance of intestinal colonisation vs. infection (translocation followed by systemic dissemination) in swine clinical cases [1]. To enlighten this controversial aspect of *S. suis* disease pathogenesis, de Greeff et al. (Appendix A) studied the putative role of intestinal colonisation for serotype 9 and showed that, during a *S. suis* serotype 9 outbreak, tonsils of piglets were colonised with different serotypes. The highest percentage of colonisation was found for serotype 9, followed by serotypes 7 and 2 (Appendix A, Table A1). Similarly, serotype 9 was the major serotype colonising the intestine (Appendix A, Table A2). The data provided by this study suggest that serotype 9 is a better coloniser of the porcine intestine than the other serotypes. Interestingly, previous in vitro studies have shown that serotype 9 isolates can adhere to porcine intestinal cells more efficiently than the other serotypes [54]. Similarly, serotype 2 strains were shown to adhere better to a human intestinal epithelial cell line than serotype 9 strains, suggesting a relationship with the high zoonotic potential of the former serotype (Appendix E). In this in vitro model, the CPS was shown to interfere with these first steps of *S. suis* colonisation and invasion, as reported in other in vitro studies with epithelial cells [56,57]. Recent developments in stem cell research allowed intestinal organoids to be used as infection models for *S. suis*. Organoids contain most cell types present in the proximal and distal intestinal mucosa and can be dissociated and grown as monolayers on semipermeable membranes [58,59]. A zoonotic *S. suis* serotype 2 strain was shown to translocate in this model, which makes it a promising in vitro model for future study of intestinal infection with *S. suis* (Appendix E).

The lack of a well-standardized in vivo mucosal infection model slowed down research progress in this area. To develop such a model, de Greeff et al. (Appendix A) applied several mucosal infection routes to piglets from different backgrounds. It was shown that intranasal inoculation in combination with oral inoculation result in a reproducible colonisation of the tonsils and the intestine of piglets; however, this mild mucosal colonisation model failed to replicate clinical disease. Different stressors facilitate and increase colonisation by *S. suis* but are not strict requirements for colonisation. A harsher mucosal challenge can be achieved by combining oral inoculation with intratracheal inoculation. In this case, the level of intestinal colonisation is lower, but more clinical symptoms like lameness, central nervous signs, or septicaemia were induced in the piglets (Appendix A). Further optimization of the oro-gastrointestinal infection model will definitively impact research aimed to dissect how *S. suis* breaches the mucosal barriers.

Encapsulated extracellular *S. suis* is a highly invasive pathogen. After penetration of host mucosal barriers, it can reach and survive in the blood and finally invades multiple organs including spleen, liver, kidney, lung, and heart. In addition, *S. suis* is able to cross the brain microvascular endothelial cells (BMECs) and/or the epithelial cells of the choroid plexus at the blood–brain barrier (BBB) and/or the blood–cerebrospinal fluid barrier to gain access to central nervous system (CNS) [56,60,61]. Septicaemia, meningitis, endocarditis, pneumonia, and arthritis are the most common forms of *S. suis* invasive disease.

The CPS of *S. suis* is without doubt the major virulence factor allowing this bacterium evasion of immune-clearance mechanisms [56,62]. However, almost all of the studies on the role of CPS used serotype 2 strains, and little is known on the role of CPSs of other serotypes as a virulence factor. Therefore, it is unknown whether differences in serotype themselves (i.e., differences in CPS structure/composition) directly affect *S. suis* virulence. To answer this question, for the first time, Okura et al. (Appendix H) experimentally generated serotype switched mutants. Six serotype switched mutants were generated using the serotype 2 reference strain P1/7 (P1/7cps2to3, P1/7cps2to4, P1/7cps2to7, P1/7cps2to8, P1/7cps2to9, and P1/7cps2to14) by exchanging the CPS synthesis gene cluster for those of serotypes 3, 4, 7, 8, 9 and 14, respectively. Their virulence was compared in mice and pigs. Only a serotype 2 switch to CPS type 4 or to type 8 showed a marked and consistent impact of bacterial virulence traits. The CPS8 conferred to *S. suis* a hyper-virulent phenotype, whereas the CPS4 conferred to *S. suis* a non-virulent character. Serotype switch from CPS2 to CPS7 or to CPS3 had restricted impact, and serotype switch from CPS2 to CPS14 or to CPS9 had no significant effect on *S. suis* virulence (Appendix H). Taken together, these findings suggest that serotype switching can differentially modulate *S. suis* virulence depending on the CPS expressed and demonstrate its importance on *S. suis* pathogenesis and clinical disease. 

Based on biochemical, bioinformatics and in vitro and in vivo gene expression studies, Ferrando et al. [63] proposed a biological model that postulates the effect of carbon catabolite repression on expression of virulence genes in the mucosa, organs, and blood. In the oropharyngeal cavity, where glucose is rapidly absorbed but dietary α-glucans persist, there is a profound effect of carbohydrate availability on the expression of virulence genes. Several virulence factors involved in adherence to host cells, degradation of connective tissue (spreading factors), and avoidance of phagocytic killing, including suilysin, are upregulated when glucose is diminished. As discussed above, suilysin may facilitate dispersion of bacteria in mucosal tissues due to loss of barrier integrity. Once *S. suis* reaches the bloodstream, metabolism is adapted for optimal growth on glucose and the expression of virulence factors is reduced. In infected organs, glucose levels are lower than in the blood and are further reduced by inflammation and utilization by *S. suis*, leading to upregulation of suilysin and other virulence factors. These studies have important implications for the design of future control strategies including the development of anti-infective strategies by modulating animal feed composition.

To further understand the genetic basis of disease in *S. suis*, Weinert et al. [64] studied the genomic signatures of human and pig clinical isolates from the United Kingdom and Vietnam. Isolates associated with disease were shown to contain substantially fewer genes than nonclinical isolates but are more likely to encode virulence factors. Human disease isolates are limited to a single-virulent population, originating in the 1920s, when pig production was intensified, but no reliable genomic differences between pig and human isolates were observed, suggesting lack of consistent genomic adaptation of *S. suis* to the human population. The authors also reported little geographical clustering of different *S. suis* subpopulations and high rates of recombination in *S. suis* worldwide, implying that an increase in virulence anywhere in the world could have a global impact over a short timescale [64].

## 4. Antimicrobial Resistance

*S. suis* infections are one of the main causes of antimicrobial usage in piglets. Indeed, the incidence of the disease may be as high as 20%, although it is usually kept lower than 5% in the field due to the extensive and routine prophylactic and/or metaphylactic use of antimicrobials. Data from antimicrobial resistance of *S. suis* worldwide are alarming, and restriction of antimicrobials as a preventive measure must be a primary concern [65]. In addition, the most effective drugs against *S. suis* are those in categories 1 and 2 (critically or highly important). The industry is trying to reduce the use of these drugs given their importance in human medicine. Indeed, *S. suis* is considered a niche for antimicrobial resistance and represents a high risk of transmission of resistance to other pathogens [65].

**Figure A1:**
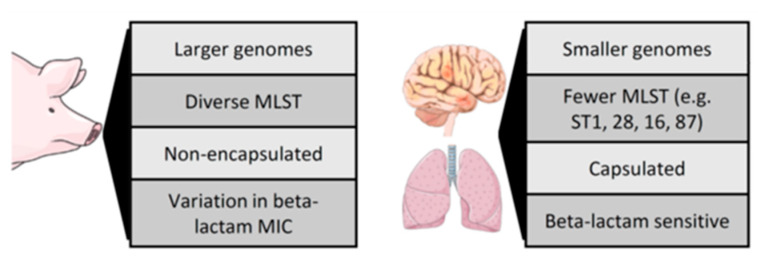
General trends in genetic and phenotypic differences between nonclinical strains of *S. suis* taken from the upper respiratory tract of pigs versus clinical strains of *S. suis* taken from the brain and lungs [64,145,146,149]: MLST = multi-locus sequence type; each MLST is defined by displaying the same allelic variant at 7 housekeeping genes. The pictures have been reproduced under a creative commons 3.0 licence from Servier Medical Art (www.smart.servier.com).

Surprisingly, despite the worldwide use of beta-lactams in pigs for over 50 years, the majority of clinical *S. suis* remains sensitive to these antibiotics. However, beta-lactam resistant strains do exist and are primarily found in commensal sites (Appendix I, Figure A1). O’Dea et al. (Appendix B) reported clinical resistance to penicillin G, albeit at a relatively low level in 8.1% of isolates. In addition, they reported similar levels of antimicrobial resistance in Australian strains compared to those overseas with regards to tetracycline (99.3%), erythromycin (83.8%), and trimethoprim/sulfamethoxazole (0.7%). Resistance to florfenicol was 14.9%, while all isolates were clinically susceptible to enrofloxacin, likely due to this being banned from use in food producing animals in Australia. Therefore, this is an aspect of *S. suis* in Australia that must be carefully monitored from both an animal and public health point of view.

In a recent work, Libante et al. [66] made a comprehensive in silico search and analysis of Integrative Conjugative Elements (ICEs) and Integrative and Mobilizable Elements (IMEs) and extensive identification of antimicrobial resistance genes present in 214 *S. suis* draft genomes. Almost 400 antimicrobial resistance genes were detected in the 214 genomes analysed. A huge amount of ICEs, IMEs, and derived elements were detected at various chromosomal sites. High diversity was observed in recombination but also in conjugation/mobilization modules. Besides ICEs, IMEs appear to be major vehicles of antimicrobial resistance genes. The authors concluded that further studies are needed to evaluate such gene fluxes inside and between ecosystems and the contribution of ICEs and IMEs in these gene transfers keeping in mind a one health global perspective.

## 5. Prevention and Control of *S. suis* Diseases

As *S. suis* is a very early coloniser of piglets, it cannot be eliminated by early weaning [1]. To reduce antimicrobial use, *S. suis* disease prevention should concentrate on management of predisposing factors and, mainly, vaccination. Despite intensive research leading to different vaccine-candidate antigens [67], no universally efficacious *S. suis* vaccine has been commercialised so far. The dream of having a universal cross-protective vaccine is highly challenging due to the high genomic diversity of *S. suis*. As discussed in the work performed by Weinert et al. (Appendix I), considerable genetic diversity of *S. suis* exists. Nonetheless, they have shown many trends in genetic and phenotypic differences between strains isolated from the upper respiratory tract of pigs without *S. suis* clinical signs (referred as “nonclinical”) and strains isolated from the lungs or systemic sites of pigs with *S. suis* clinical signs (referred as “clinical”) (Appendix I, Figure A1). Overall, the genetic diversity of clinical *S. suis* is less than that of nonclinical strains, implying that a design of a universal vaccine to control *S. suis* clinical infection might be possible. 

Over 38 subunit vaccine candidates have been reported [67,68] and new ones are continuously being characterised, yet homologous protection is still controversial and cross-protection (either against other serotypes or at least using heterologous strains) was evaluated in few of these studies [69,70,71,72,73,74,75]. Lack of well-standardized animal models for immunization and challenge, especially for serotypes other than 2, is one of the reasons of limited progress towards a subunit vaccine, which also results in contradictory results for a same vaccine candidate [67]. Amongst other confounding factors are the use of mouse models (for pre-screening) without confirmation in swine trials, number of vaccine doses, and different adjuvants (which are not always compatible with a future use in swine medicine). More research, with better standardized protocols, would certainly advance subunit vaccine development. 

One common vaccine target is, without doubt, the CPS because the majority of clinical strains have a capsule (Appendix I, Figure A1). Nevertheless, the vaccine-induced protection will be serotype-restricted. Multiple different polysaccharide epitopes should be selected in a vaccine to target different serotypes, and vaccination-driven strain replacement of the population might be expected. Finally, in addition to their diversity and as aforementioned, *cps* loci are often recombination “hot-spots” and this gives rise to CPS “switching” between strains. Highly conserved proteins might help overcome these limitations and fight strain replacement/evolution. As *S. suis* is part of the commensal flora, any preventive/control strategy should eliminate clinical isolates without altering the precious balance of the pig mucosal microbiota. Albeit possible disease-protective limitations, a systemic vaccination approach is to be prioritized in this context (Appendix I).

Due to the young age of the piglets affected and the lack of an effective commercial vaccine (as discussed above), many farms use metaphylactic perinatal antimicrobials to control *S. suis* disease. Besides the public health concern related to antimicrobial use in production animals and the increase in antimicrobial resistance, an additional problem of antimicrobial usage is that these treatments can also affect the beneficial bacteria of the microbiota. In an attempt to understand the harmful effect of overuse of antimicrobials at early age, Aragon et al. (Appendix J) have evaluated the outcome of perinatal antimicrobial treatment on the nasal microbiota at weaning. Elimination of perinatal antimicrobials resulted in an increase in bacterial diversity in the nasal microbiota at weaning. Furthermore, management of lactation without perinatal antimicrobials had a beneficial impact later in life in terms of animal health and productivity in the nursery phase. Albeit more detailed studies are required, Aragon et al. also observed that perinatal ceftiofur administration might favour colonisation with potentially clinically relevant serotypes of *S. suis*. As such, the precise effects of antimicrobial metaphylaxis are difficult to predict (Appendix J).

The only available vaccines used in the field are autogenous, which consist of killed bacteria (“bacterin”) from the predominant strain(s) recovered in an affected farm, produced by licenced laboratories and given back to the same farm only. However, there are very few scientific studies demonstrating whether the use of such vaccines in the field correlates with a reduced mortality and curative antimicrobial use. Indeed, field peer-reviewed reports on autogenous vaccines are almost nonexistent (only 2 published papers in the last 30 years) [76,77]; others are incomplete and, in most of them, a control (non-vaccinated) group is missing, which may preclude any scientifically sound conclusions [68]. On the other hand, controlled experimental (laboratory) studies have shown contradictory results concerning bacterin-induced protection [67,68]. That controversial protective response could be attributed, among others, to a loss of antigenicity caused by the killing procedure and/or production of antibodies against antigens not associated with protection. Moreover, autogenous vaccines are “manufacturer-related” (each licenced laboratory uses different protocols, antigen concentration, adjuvants, etc.) and “farm-specific” (useful against homologous—same strain—challenge only) [67]. Finally, the correct diagnosis of *S. suis* as a primary cause of disease may complicate the choice of the strain(s) to be included in the autogenous vaccine. There is still an unresolved issue concerning isolates recovered from lungs, which are considered by many researchers as secondary invaders and may or may not (depending on the laboratory) be included in the vaccine as antigens. Definitively, more studies are required to generate scientific knowledge to improve this important preventive tool and to help reduce the use of antimicrobials. 

## 6. Policy on Autogenous Vaccine Manufacturing

The wide use of autogenous vaccines and cross-border movement of animals vaccinated with autogenous vaccines is now a common practice within Europe. Therefore, harmonising quality of these products has been considered necessary by the Coordination Group for Mutual Recognition and Decentralised Procedure—Veterinary (CMDv) and the national competent authorities for Veterinary Medicinal Products (VMPs) in Europe. M. Saléry discussed in Appendix K the regulatory framework of the manufacture, control, and use of autogenous vaccines within the European Union. Recently, a new legislation on VMPs has been adopted in European Union, applicable in January 2022. In this new regulation, autogenous vaccines have an updated definition and fall under European regimen. Article 2(3) of this regulation requires that some articles of the regulation apply to “inactivated VMP manufactured from pathogens and antigens obtained from an animal or animals in an epidemiological unit and used for the treatment of that animal or those animals in the same epidemiological unit or for the treatment of an animal or animals in a unit having a confirmed epidemiological link”. The introduction of the concept of epidemiological link allows now the use of autogenous vaccines in parental lines or for animals prior their introduction in fattening sites where they will be in contact with new pathogens. In addition, different laboratories working under different conditions presently exist in Europe. From January 2022, all autogenous vaccines will have to be done under Good Manufacturing Practices (GMP) or GMP-like requirements. The manufacturers will have to be authorized, and the compliance with the requirements will be controlled by inspection. As a consequence, autogenous vaccines will be produced under similar quality conditions. However, among others, differences in antigen preparation, antigen concentration, method of killing, and type and concentration of the adjuvants will still exist. In Appendix K, M. Saléry summarized key points from the CMDv’ recommendation paper [78], which deals with the manufacture, the control, and the use of viral and bacterial autogenous vaccines in Europe. In the framework of the new regulation, the CMDv’ recommendations will be updated in the coming years. The current and future regulatory framework ensures those products are of high quality. Nevertheless, safety and efficacy are not regulated. Safety and, more importantly, efficacy of *S. suis* autogenous vaccines need to be better demonstrated through field use and laboratory research.

## 7. Conclusions

Intensification of animal food production and emergence of new production systems (such as raised without antibiotics or organic systems as well as “Humane” animal farming) has resulted in emergence or reemergence of pathogens. In this context, coinfections are more likely to occur, increasing the incidence and/or enhancing clinical disease with certain pathogens, including *S. suis*. Continued development of suitable models for polymicrobial infections and a better understanding of the underlying pathological mechanisms are required to develop effective intervention strategies to prevent the effects of these diseases on swine production.

Mucosal infection models still require optimization and have the potential to improve our knowledge of the pathogenesis of *S. suis*-induced disease. These models would significantly contribute to vaccine development as well. Research on mucosal infection and immunity will also help address the potential use of nutrition management and/or microbiota enhancement to improve swine health and, consequently, *S. suis* control strategies. Early medication with antimicrobials has to be carefully considered since antimicrobials may interfere with the establishment of the microbiota and, in consequence, with immune maturation and other microbiota functions, which may have a lasting health effect later in life.

*S. suis* importance as a zoonotic pathogen is continuously increasing due to, at least in part, the pressure to reduce antimicrobial use in livestock. This public health threat is enhanced by the lack of universally effective vaccines that might reduce the infection load in swine and thus the risk for humans. Global strengthening of swine trade amongst countries has the potential to facilitate exchange of genetic material through recombination and mobile genetic elements, which may result in selective advantage and niche adaptation. It is crucial to understand the factors that are involved in the ability to cause zoonotic infection and to monitor if these may eventually result in *S. suis* strains that are more human adapted. 

Indeed, a better worldwide surveillance system of *S. suis*-related disease in swine and humans would improve our understanding of the epidemiological evolution of this pathogen. New molecular tools have been developed; however, due to the diversity of *S. suis* and lack of appropriate markers to differentiate virulent strains from commensal ones, alternative techniques might still be required to achieve a comprehensive understanding of the *S. suis* bacterial community, including virulence-associated gene profiling. The development of such tools that might eventually allow prediction of virulence potential of a strain might be compromised by sampling strategies and the rich *S. suis* community in the upper respiratory tract of healthy animals.

In the era of antimicrobial restrictions and new social meat consumption trends, improvement and/or development of vaccination strategies is of utmost importance. In spite of decades of research on *S. suis* vaccines, autogenous bacterins are the only control strategy available that swine producers have access to. Due to intensive use of these vaccines in Europe, new policies are in place that would improve and normalize their manufacturing. The implantation of similar policies worldwide, including North America, where the use of autogenous vaccines is also widespread, would be expected or sought after. Nevertheless, more field studies are essential to scientifically validate their protective effect and, consequently, their cost–benefit impact for swine producers. 

## Figures and Tables

**Figure A2:**
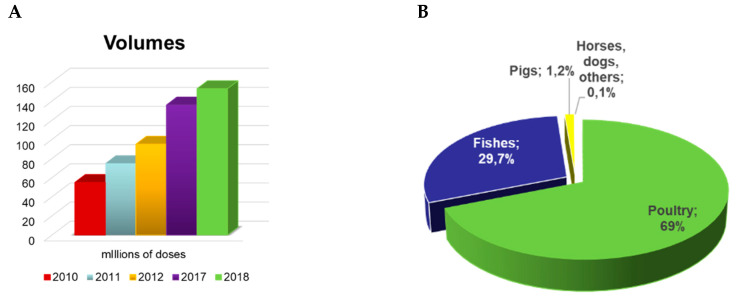
Volume of autogenous vaccine doses between 2010 and 2018 (**A**) and animal production distribution in France (**B**). Source: French agency for food, environmental, and occupational health safety (ANMV) data.

## Appendix A

**Proceeding from the 4th International Workshop on *Streptococcus suis***

*Streptocccus suis* serotype 9 infection: Novel animal models and diagnostic tools

Astrid de Greeff ^1^, Xiaonan Guan ^2^, Francesc Molist ^2^, Manon Houben ^3^, Erik van Engelen ^3^, Ton Jacobs ^4^, Constance Schultsz ^5^, Kees van der Ark ^5^, Helmi Fijten ^1^, Hilde Smith ^1^ and Norbert Stockhofe-Zurwieden ^1^

^1^ Wageningen Bioveterinary Research, Lelystad, The Netherlands
^2^ Schothorst Feed Research, Lelystad, The Netherlands
^3^ Animal Health Service, Deventer, The Netherlands
^4^ MSD Animal Health, Boxmeer, The Netherlands
^5^ Amsterdam Institute for Global Health and Development, Amsterdam, The Netherlands

**A.1. Serotype 9 is the dominant serotype in Western Europe**

*Streptococcus suis* is a highly diverse organism; currently 29 serotypes have been described [6,29]. Historically, serotype 2 was the predominant serotype causing infections in piglets worldwide. However, recently, a shift towards serotype 9 has occurred, in particular in Western Europe [2]. In recent years, however, incidence of serotype 9 infections in pigs increased in South East Asia [79] and to a lesser extent in North America [80]. In Thailand, serotype 9 was isolated from a human patient with severe disease symptoms [24], emphasizing the zoonotic potential of serotype 9 isolates, that was already suggested [26].

In The Netherlands, all farms are colonised by serotype 9. Colonisation with *S. suis* serotype 9 is not directly correlated with *S. suis* disease. Obviously, additional risk factors are determinative in causing disease. Unfortunately, autovaccination against serotype 9 is less effective than against serotype 2, making it difficult to combat the infection [81,82]. Under experimental conditions, *S. suis* serotype 9 is hardly virulent [83], whereas in practice, many piglets develop severe invasive disease after *S. suis* serotype 9 infection. There could be different explanations for this discrepancy. First of all, additional risk factors occurring under field conditions may play a role in disease development like existing viral or bacterial coinfections, management factors, or housing conditions. Alternatively, serotype 9 isolates in the field might differ in virulence from the isolates used in experimental infections. It has been described that the *S. suis* serotype 9 population is genetically very diverse [10,64,79,84]. In addition, it has been described that differences in virulence exist within the serotype 9 population [79], similar to that described for serotype 2 [85].

Taken together, *S. suis* serotype 9 has been emerging for the last decade and is spreading around the world. Due to the large genetic and phenotypic heterogeneity in the serotype 9 population, it is a complex pathogen to study.

**A.2. Population genetics of *S. suis* serotype 9**

To get more insight in the genetic population of *S. suis* serotype 9 in The Netherlands, an epidemiological study was performed [8]. Two populations of serotype 9 isolates were included for genotyping analysis: clinical isolates and isolates from healthy carrier piglets. Seventy-eight isolates from clinical cases of *S. suis* were included in the study and were isolated from the CNS of animals with invasive *S. suis* disease (meningitis). The isolates from healthy carrier pigs were isolated from 6 independent farms that did not have clinical outbreaks caused by *S. suis* for at least two years (n = 50). Tonsils of healthy sows and piglets were sampled, and *S. suis* serotype 9 isolates were isolated after plating. Genotyping of the isolates using Comparative Genome Hybridization (CGH) revealed that the two populations were genetically very distinct. CGH split the population in two clusters: one cluster only contained carriage isolates, whereas the other cluster contained all clinical isolates plus a few carriage isolates. These carriage isolates probably reflect clinical isolates that are not causing any clinical outbreaks on the farms but do have virulent potential.

To further analyse the genetic differences between the isolates, all serotype 9 isolates were subjected to whole genome sequencing. A systematic analysis of the data showed that the clinical isolates clustered mainly together in one group whereas the carriage isolates were distributed across 8 different groups. This suggested that virulent serotype 9 isolates are genetically very similar, whereas the carriage isolates are more heterogeneous [8], as was described before [10,64,79,84]. Based on our data, the heterogeneity was only found for the carriage isolates.

Our data suggest that at least two virulence phenotypes of serotype 9 isolates exist: clinical isolates that are virulent and avirulent carriage isolates. To confirm there are differences in virulence among serotype 9 isolates, four isolates were tested for virulence under experimental conditions. Two carrier isolates, one clinical isolate, and one laboratory strain that clustered among the clinical isolates were tested for virulence by infecting CDCD piglets intravenously with a high dose of each of the isolates. The survival curves clearly showed there was a significant difference in virulence between carriage isolates and clinical isolates. Although the carriage isolates could induce *S. suis* specific disease symptoms like lameness, 80%–90% of the piglets survived the infection, whereas the piglets infected with the clinical isolates all died within 2 days postinfection. In conclusion, it was shown that, within *S. suis* serotype 9, the virulence of clinical isolates and tonsillar carriage isolates differed significantly.

**A.3. Intestinal translocation**

Due to the zoonotic *S. suis* outbreaks in South East Asia, a lot of attention has been paid to putative risk factors for human *S. suis* infections [19,86]. One of the risk factors that has been identified is occupational exposure, which is the main risk factor for human infections in the Western world as well [87,88]. However, a new risk factor that was identified due to the outbreak in Asia is consumption of raw or undercooked pig products like raw blood soup. By ingesting contaminated pork products, *S. suis* infections can occur. This insight directed research into the role of intestinal translocation in porcine *S. suis* infections.

It is known that *S. suis* colonises the gastrointestinal tract of piglets in high numbers, in particular after weaning [89]. In addition, under experimental conditions, invasive *S. suis* serotype 2 infections could be induced by intestinal inoculation of piglets [55]. To study the putative role of intestinal colonisation for serotype 9, an epidemiological study was performed at the Wageningen University and Research experimental farm in Sterksel, The Netherlands. It was shown that, during a *S. suis* serotype 9 outbreak, tonsils of piglets were colonised with different serotypes. The highest percentage of colonisation was found for serotype 9, followed by serotype 7 and 2 (Table A1). Only a few animals were colonised by serotype 1.

Subsequently, colonisation in *S. suis* specific organs and the intestine was studied in 20 *S. suis* diseased animals and 20 healthy litter mates. It was shown that specific organs were exclusively colonised by serotype 9, as was expected since there was a serotype 9 disease outbreak on the farm. However, up to 85% of the healthy controls and 95% of the diseased piglets were also colonised by serotype 9 in the intestine, whereas the other serotypes did not colonise at all in the intestine or only in a limited number of animals (Table A2). Faecal samples were also screened for presence of serotypes 1, 2, 7, and 9. Whereas none of the piglets were positive for serotypes 1 or 2, a few animals were positive for serotype 7 and a considerable number of animals were positive for serotype 9 in faeces.

**Table A1:** Presence of *S. suis* serotypes 1, 2, 7, and 9 in tonsils and faecal samples of piglets at Sterksel farm as determined using qPCR.

Sample	Time Point	*S. suis* 1	*S. suis* 2	*S. suis* 7	*S. suis* 9
Tonsil	1 day preweaning	10/132 (8%)	7/132 (5%)	43/132 (33%)	113/132 (86%)
	1 week postweaning	2/130 (2%)	10/130 (8%)	80/130 (62%)	124/130 (95%)
	4 weeks postweaning	3/109 (3%)	19/109 (17%)	80/109 (73%)	105/109 (96%)
Feces	1 day preweaning	0/132 (0%)	0/132 (0%)	1/132 (1%)	45/132 (34%)
	1 week postweaning	0/130 (0%)	0/130 (0%)	3/130 (2%)	31/130 (24%)
	4 weeks postweaning	0/109 (0%)	2/109 (0%)	0/109 (0%)	11/109 (10%)

**Table A2:** Presence of *S. suis* serotypes 1, 2, 7, and 9 in specific organs (joints and central nervous system (CNS)) and intestinal samples of piglets at Sterksel farm as determined using qPCR.

Sample	Status	*S. suis* 1	*S. suis* 2	*S. suis* 7	*S. suis* 9
Joints/CNS	Diseased	0/20 (0%)	0/20 (0%)	0/20 (0%)	16/20 (80%)
	Healthy controls	0/20 (0%)	0/20 (0%)	0/20 (0%)	0/20 (0%)
Intestine	Diseased	0/20 (0%)	0/20 (0%)	5/20 (25%)	19/20 (95%)
	Healthy controls	0/20 (0%)	1/20 (5%)	5/20 (25%)	17/20 (85%)

Although only one farm was sampled in this study, the data suggest that serotype 9 is a better coloniser of the porcine intestine than the other serotypes. This suggestion was supported by the data published by Ferrando et al. [54] that showed that serotype 9 isolates can adhere to porcine intestinal cells more efficiently that the other serotypes. Furthermore, it was shown that translocation of *S. suis* serotype 2 via the intestine can cause severe invasive disease in piglets.

**A.4. Mucosal infection model**

The putative role of the intestine in invasive translocation of *S. suis* opens up new possibilities in the prevention of *S. suis* disease. If the intestine plays an important role in the pathogenesis of disease, feed interventions might contribute to prevention of disease. Feed components can either strengthen the intestinal barrier thus preventing translocation, they can exhibit antimicrobial effects and kill *S. suis*, or they can affect microbiota composition in such a way that *S. suis* cannot colonise anymore (competitive exclusion). To evaluate the effect of this kind of interventions, an experimental mucosal infection model is required. At the moment, only an intravenous model is available that leads to severe invasive disease symptoms. A more subtle infection model following mucosal challenge route would be very interesting.

To develop such a model, several mucosal infection routes were applied to piglets from a different background. It was shown that intranasal inoculation in combination with oral inoculation will result in a reproducible colonisation of the tonsils and the intestine of piglets, even when they are endemically colonised by serotype 9. To mimic the weaning stress piglets experience under field conditions, several stressors were applied to the piglets, like transport stress, social stress (no acclimatization), or pretreatment of the nasal mucosa with acetic acid. These stressors facilitate and increase colonisation by *S. suis* but are a not strict requirement for colonisation. This mild mucosal colonisation model does not lead to clinical disease. A harsher mucosal challenge can be achieved by combining oral inoculation with intratracheal inoculation. In this case, the level of intestinal colonisation is lower, but more clinical symptoms like lameness, central nervous signs, or septicaemia were induced in the piglets.

Before applying any of those models, some critical parameters need to be determined. First of all, the background of the piglets is of utmost importance for the outcome of the experiment. Piglets from the field with maternal antibodies and a natural exposure to endemic pathogens are more resilient to experimental *S. suis* infection. This is particularly true for animals from farms with a relative high hygiene standard and good management practices. Piglets from high health farms with a specific pathogen free status are more susceptible to infection. CDCD piglets are very sensitive to all infections due to their complete naive immune system. Another important parameter is the application of a stressor. Especially when more resilient piglets are used, a stressor is very helpful to increase susceptibility to colonisation and disease. Several stressors can be used; in our studies, social stress, transport stress, and acid exposure were applied.

**A.5. Conclusions**

In conclusion, it was shown that *S. suis* serotype 9 is a very heterogeneous pathogen that has at least two pathotypes. Carriage isolates are very diverse and have a low virulence, whereas clinical isolates are genetically less diverse and more virulent (though they are less virulent than MRP+EF+ serotype 2 isolates). New pig models were developed for experimental colonisation and disease caused by *S. suis* serotype 9. These models can be used for intervention studies aiming to reduce colonisation or disease caused by *S. suis* serotype 9. There are strong indications that intestinal colonisation plays an important role, in particular, for serotype 9 infections. More research is required to study this *porte d’entrée* in more detail.

## Appendix B

**Proceeding from the 4th International Workshop on *Streptococcus suis***

The genomics of Australian *Streptococcus suis*: where do we fit in the global scene?

Mark A O’Dea ^1^, Tanya Laird ^1^, Rebecca Abraham ^1^, David Jordan ^2^, Kittitat Lugsomya ^3^, Laura Fitt ^4^, Marcelo Gottschalk ^5^, Alec Truswell ^1^, and Sam Abraham ^1^

^1^ Antimicrobial Resistance and Infectious Disease Laboratory, School of Veterinary and Life Sciences, Murdoch University, Perth, Western Australia, Australia
^2^ Wollongbar Primary Industries Institute, NSW Department of Primary Industries, NSW, Australia
^3^ Department of Veterinary Microbiology, Faculty of Veterinary Science, Chulalongkorn University, Bangkok, Thailand
^4^ ACE Laboratory Services, Bendigo, Victoria, Australia
^5^ Faculty of Veterinary Medicine, University of Montreal, Saint-Hyacinthe, Quebec, Canada

Information relating *to Streptococcus suis* in the Australian pig herd is limited [11,12]. Given the paucity of data available on *S. suis* associated with disease in Australian pigs, the aim of this study was to analyse and characterise a significant number of *S. suis* isolates from diseased pigs obtained across multiple production sites and spanning a seven-year period.

A total of 148 swabs from archived clinical isolates spanning the years 2010 to 2017 were subjected to antimicrobial susceptibility testing via broth microdilution; whole genome sequencing via Illumina Nextseq; and detailed genomic interrogation for presence of antimicrobial resistance elements, putative virulence genes, serotype, and MLST. In addition, core genome phylogenetic analysis of Australian isolates and 383 previously published international isolates was performed.

Genomic analysis of the 148 isolates revealed that 110 isolates belonged to 11 previously identified MLSTs, with 38 isolates belonging to one of 26 newly identified MLSTs. The most prominent MLSTs were ST 27 (27/148), ST 25 (26/148), ST 28 (11/148), ST 483 (11/148), and ST 1 (10/148).

Analysis of the capsular genes resulted in two major serotypes being detected: serotype 2 (39/148) and serotype 3 (37/148). The other main serotypes were 1/2 (11/148), 16 (8/148), 19 (8/148), 8 (7/148), and 4 (7/148). Analysis of serotype and MLST combinations showed a high proportion of isolates as serotype 2 ST25 (17.6%), serotype 3 ST27 (18.2%), and serotype 2 ST28 (6.1%).

The proportions of isolates carrying the main virulence factors *mrp*, *epf*, or *sly* across all isolates were 7.4% (*cps* 1/2, 2, 14, and 19), 6.8% (*cps* 1/2, 2, and 14), and 33.1% (*cps* 1/2, 2, 3, 4, 5, 7, 8, 11, 12, 14, 15, 16, 18, 19, 21, and 23), respectively. Only a single isolate carried both *mrp* and *sly*, while 6.8% of isolates carried both *mrp* and *epf* and 6.8% carried *mrp*, *epf*, and *sly*. The isolates carrying all three genes belonged to serotypes 1/2 (n = 7), 2 (n = 2), and 14, and all belonged to ST1, with a significant association between ST1 and the carriage of all three genes (Fisher’s exact test *p*-value < 0.0001).

Detailed examination of the virulence gene profiles demonstrated that the Australian isolates could be grouped in multiple clades based on putative virulence gene carriage. Of most significance was a single clade containing a conserved block of 32 putative virulence genes, which was the only group to contain the genes *dppIV*, *epf*, *mrp*, *sbp2*, *oppA*, and *lde*. This group consisted predominantly of serotype 1/2 and serotype 2 isolates, and all were ST1.

On average, the total gene content of systemic isolates from brain and joint was 2057, significantly lower than that from respiratory isolates which carried 2109 (Mann–Whitney U test *p*-value = 0.025). When the total gene content of isolates was compared to the number of putative virulence genes, a trend was observed based upon ST. This was most apparent in the ST1 isolates which had a significantly higher number of putative virulence genes compared to the other STs and the lowest average total gene content of 1918 (*p*-value < 0.00001).

Phylogenetic comparison of the *S. suis* strains isolated from Australian pigs against a global collection of *S. suis* strains resulted in the identification of four major clades. The strains from this study were present in all four clades. The majority of the Australian strains (89/148) clustered in clade 2, which was comprised entirely of strains from the UK, North America, and Canada. Only eight strains were present in clade 4, which was made up of Vietnamese and UK isolates. While Australian serotype 2 ST25 isolates could be distinguished from North America and Canadian strains, the small SNP difference was notable; as aside from a single isolate, Canadian and Australian isolates differed by a maximum of only 100 SNPs across the core genome.

A high proportion of the isolates were resistant to both tetracycline (99.3%) and erythromycin (83.8%). Low levels of resistance were observed for florfenicol (14.9%), penicillin G (8.1%), ampicillin (0.7%), and trimethoprim/ sulphamethoxazole (0.7%). None of the isolates were resistant to enrofloxacin.

In this study, we present the key findings of the virulence potential of ST1 clones and the limited evolution of Australian clones from their global seed strains. In terms of virulence genes, the *mrp*+/*epf*+/*sly*+ combination was carried by all ST1 isolates, and these were consistently the isolates with the largest array of putative virulence factors. In addition, a conserved constellation of virulence genes, a number of which are associated with adhesion and which were not present in other MLSTs, provides some insight into the virulence of ST1 clones. When this gene block was investigated in ST1 clones from overseas, it was also found to be highly conserved across 226 isolates. Taken together, these factors suggest that both Australian and international ST1 clones have a distinct fitness advantage in terms of binding ability towards host target cells, and ST1 bacterial adhesion factors may be a promising vaccine target.

Antimicrobial resistance of Australian strains was similar to levels reported overseas with regards to tetracycline (99.3%), erythromycin (83.8%), and trimethoprim/sulfamethoxazole (0.7%). Resistance to florfenicol was 14.9%, while all isolates were clinically susceptible to enrofloxacin, likely due to this being banned from use in food producing animals in Australia. Of concern was the observed clinical resistance to penicillin G, albeit at a relatively low level in 8.1% of isolates. Therefore, this is an aspect of *S. suis* in Australia that must be carefully monitored from both an animal and public health point of view.

In conclusion, our findings demonstrate that Australian clones of *S. suis* associated with clinical disease in pigs have maintained a stable core genome, mirroring the international seed-stock from which they were derived. Australian clones associated with disease in pigs consist predominantly of serotypes 2, 3, and 1/2, which is consistent with reports from other pig producing countries [2]. Despite the limited number, the characterisation of serotype 1/2 ST1 clones is significant, as all displayed distinctive factors associated with highly virulent *S. suis*.

## Appendix C

**Proceeding from the 4th International Workshop on *Streptococcus suis***

Characterisation of *Streptococcus suis* isolates from the United States by serotyping, MLST, and virulence-associated gene profiling

Connie Gebhart ^1^, April Estrada ^1^, Marcelo Gottschalk ^2^, Aaron Rendahl ^1^, Douglas Marthaler ^3^

^1^ College of Veterinary Medicine, University of Minnesota, USA
^2^ Faculty of Veterinary Medicine, University of Montreal, Canada
^3^ Veterinary Diagnostic Laboratory, College of Veterinary Medicine, Kansas State University, USA

*Streptococcus suis*, an increasingly important pathogen of swine in the United States of America (USA), is composed of both pathogenic and commensal strains. Commensal strains normally reside in the upper respiratory tract of pigs, and pathogenic *S. suis* strains are associated with meningitis, arthritis, endocarditis/epicarditis, polyserositis, and septicaemia in piglets and growing pigs [1,90]. Identifying major disease-causing strains can promote the development of treatment and control plans.

MLST is a widely used method for subtyping strains, but information on the subtype distribution of *S. suis* strains in the USA is limited or outdated. A 1992 study investigated the serotype distribution of porcine *S. suis* strains in Minnesota and reported the prevalence of serotypes 2–9 and 11, of which serotype 2 was the predominant serotype associated with neurological disease [91]. A 1993 study identified serotypes 1–8 and 1/2 in naturally infected pigs, with serotype 2 being the predominant serotype in USA [92]. A large USA study in 2009 investigated the serotype distribution of *S. suis* strains collected from 2003 to 2005 from 17 states. This 2009 study illustrated that the distribution of strains was similar to that in Canada in which serotypes 1/2, 2, 3, 7, and 8 were most prevalent in diseased pigs [93,94] and dissimilar to the distribution in Europe in which serotype 2 occurs at a considerably higher percentage of isolates than in North America [95]. On the other hand, studies addressing MLST for *S. suis* largely focus on common STs of serotype 2. ST25 and ST28 are more common among strains recovered from diseased animals in North America, while ST1 strains are more prevalent in Europe and Asia [25,83,96].

The objective of this study was to characterise the diversity of 208 *S. suis* isolates collected between 2014 to 2017 across North America (mainly the USA) by serotyping and MLST to address the limited information on current *S. suis* strains circulating within the USA [10]. Isolates were also characterised by pathotype, based on clinical information and site of isolation, as pathogenic (from neurologic or systemic tissues, n = 139), possibly opportunistic (from lung samples, n = 47), or commensal (from healthy pigs, n = 22). The subtype distributions were further analysed by pathotype to determine prevalent subtypes recovered from diseased pigs.

Serotyping identified 20 different serotypes and the predominant serotypes were 1/2 (n = 54) and 7 (n = 23). Fifty-eight different STs were identified and 38 were newly identified STs (961–969, 971–998, and 1001; n = 56). The predominant ST was ST28 (n = 52), followed by ST94 (n = 18), ST1, and ST108 (n = 17 each). Fifteen of the 20 serotypes identified contained multiple STs, with the number of different STs within a single serotype ranging from 2 to 8. The subdivision of *S. suis* serotypes by ST further illustrated the high diversity among the isolates. Furthermore, the isolates were selected from 20 different states, representing the major swine producing states in the USA. Geographic distribution of the *S. suis* serotypes in our study identified serotype 1/2 in 13 of the 20 states, with a concentration in five.

We investigated associations between subtype (serotype and MLST) and pathotype classifications using odds ratio (OR) and phylogenetic analyses. Between 80%–100% of isolates belonging to serotypes 1, 1/2, 2, 7, 14, and 23 were classified as the pathogenic pathotype, and these associations were supported by OR analysis. Twelve STs, including STs 1, 28, 94, and 108, contained over 75% of isolates classified as pathogenic, which demonstrated the same associations by OR. Interestingly, many of the novel STs that occurred as singletons were identified as the commensal pathotype. Commensal isolates are not generally subjected to subtyping by MLST, which may explain the large number of novel STs.

To investigate genetic relationships among the samples and possible associations among serotype, genotype, and pathotype classifications, the MLST allelic sequences were clustered. MLST clustering analysis demonstrated three clades with an association to pathotype. The first clade represents mostly the pathogenic pathotype and includes largely serotype 1 (with some serotype 2 and 14) and ST1 isolates. The second clade represents mostly the pathogenic pathotype and contains serotype 1/2 and ST28 isolates, the predominant subtypes in our USA sample set. The third clade represents mostly the commensal pathotype and a variety of serotypes and STs (most of them novel STs).

These results demonstrated the use of serotyping and MLST to differentiate pathogenic from commensal isolates and to establish links between pathotype and subtype, thus increasing the knowledge on *S. suis* strains circulating in the USA. However, due to the diversity of *S. suis*, alternative subtyping techniques should be explored to consistently differentiate pathogenic from commensal strains. Alternative subtyping techniques include virulence-associated gene (VAG) profiling of *S. suis*. Identification of the virulence factors of *S. suis* has been previously explored for understanding the pathogenesis of *S. suis* infection, and there are currently over 100 putative virulence factors reported [3,56,84,97]. However, information on the distribution of these factors in USA isolates is limited.

We characterised the same set of 208 isolates by VAG profiling to investigate the distribution of the 66 VAGs in USA isolates. Presence/absence of these 66 VAGs was used to perform clustering analysis, which also demonstrated three clades with an association to pathotype; these clades were further investigated by serotype and ST. The first clade represents the pathogenic pathotype containing serotypes 1 and 2 and ST1, consistent with publications associating ST1 with high virulence [96,97]. The second clade largely represents the pathogenic pathotype and contains isolates subtyped as serotype 1/2 and ST28. The third clade contains isolates of the commensal and possibly opportunistic pathotypes belonging to numerous serotypes and STs. The three classical VAGs of *S. suis* serotype 2 strains (*epf*, *sly*, and *mrp*) were found in the ST1 clade but not in the ST28 clade. Several other VAGs were identified that may potentially differentiate the pathogenic from the commensal pathotypes and may be better predictors of pathogenicity for USA strains.

In this study, 208 *S. suis* isolates from North America were characterised by serotyping, MLST, and VAG profiling. These findings will contribute to the knowledge on the population structure of *S. suis* strains currently circulating in the USA. We demonstrated the use of serotyping and MLST for the subtyping of strains and for investigating associations between subtypes and pathotype to identify pathogenic strains. The identification of pathogenic strains is important for enhancing strain selection for preventive strategies. The subtype distributions in our study elucidated the predominance of serotype 1/2 ST28 and ST1 isolates from clinically affected pigs in the USA, which may inform new diagnostic approaches and virulence studies. We also determined the diversity of the isolates by MLST and VAG clustering analyses, both of which illustrated one commensal and two pathogenic clades [10].

## Appendix D

**Proceeding from the 4th International Workshop on *Streptococcus suis***

Situation of human *Streptococcus suis* infections in Thailand

Anusak Kerdsin ^1^

^1^ Faculty of Public Health, Kasetsart University Chalermphrakiat Sakon Nakhon Province Campus, Sakon Nakhon, Thailand

In Thailand, *S. suis* infection was first described in 1987, with 2 cases of meningitis [98]. Since the largest outbreak of human *S. suis* infection occurred in Sichuan province, China in 2005 [16], the importance of this disease has been increasingly recognized in Thailand. Up to the present, 4 outbreaks of *S. suis* infections in humans have been recorded in Thailand. The first outbreak including 29 laboratory confirmed cases and 3 deaths, occurring in Phayao Province during May of 2007 [99]. A second outbreak was recognized in Chiang Mai and Lamphoon provinces during June–July, 2008, including 44 confirmed cases, 26 suspected cases, and 3 fatal cases with septic shock (http://www.boe.moph.go.th/Annual/Annual%202552/WESR2552/wk52_38/wk52_38.pdf). The third outbreak was found in Phetchabul province in April, 2010; this outbreak had 14 confirmed cases with 5 fatal (http://www.boe.moph.go.th/Annual/aesr2553/wesr_2553/wk53_17.pdf). The fourth outbreak has been reported in Uttaradit province in May, 2019 with 14 cases (https://ddc.moph.go.th/uploads/files/67f5dfba9522d8862ca862d9c43a5a66.pdf).

Consequently, a total of 2011 cases and 131 fatal cases of *S. suis* infection in Thailand have been reported by the Bureau of Epidemiology, Ministry of Public Health since 2013–August, 2019 (http://www.boe.moph.go.th/boedb/surdata/disease.php?dcontent=situation&ds=82). Of note, case fatality rate was 6.5% in contrast with 2 retrospective studies that revealed case fatality rates of 9.5% and 12.7%, respectively [100,101]. However, a prospective study demonstrated a case fatality rate of 16.1% and an incidence rate of 6.2 per 100,000 in the general population. The main route of *S. suis* infection in Thailand was consumption of traditional raw pork or pig’s blood products [99,15,100,101]. Food safety campaign implementation in Phayao province during 2011–2013 showed a marked decrease of the incidence proportion [20]. The annual incidence proportion significantly decreased from 6.4/100,000 persons in 2010 (before implementation) to 2.7/100,000 persons in 2011, to 2.0/100,000 persons in 2012, and to 3.5/100,000 persons in 2013. This revealed a 3.94/100,000 persons decrease in the trend of incidence proportion after campaign (*p* < 0.001) [20].

Microbiological characterisation of *S. suis* in Thailand showed that serotype 2 (93.4%) was the main serotype in human infections in Thailand, followed by serotype 14 (5.2%), 24 (0.6%), 5 (0.4%), 4 (0.1%), 9 (0.1%), and 31 (0.1%), respectively [14,22,24,100,101,102,103]. MLST classified serotype 2 into 5 clonal complexes (CC) that were CC1, CC25, CC28, CC104, and CC233/379. While serotype 14 was classified to only CC1 by MLST, ST1 was the major ST of serotype 2 found in CC1, whereas ST105 was the main ST in CC1 of serotype 14. ST104, ST25, ST28, and ST233 were the main STs in CC104, CC25, CC28, and CC233/379 of serotype 2 [14,22,100,101]. Of note, ST1 and 104 (serotype 2) are predominant STs in Thai human infections. In contrast, serotypes 24 and 31 and some isolates of serotype 5 were classified in CC221/234, with the majority belonging to ST221. It is also interesting that CC104, CC233/379, and CC221/234 are exclusively found in Thailand only. MLST determined that the Thai serotype 9 isolates were of ST16, which belongs to clonal complex 16 [24]. Serotype 9 is the most common serotype affecting pigs in European countries, especially ST16 which is found in ≥89% of cases in The Netherlands [2,25].

## Appendix E

**Proceeding from the 4th International Workshop on *Streptococcus suis***

Understanding the emergence of zoonotic *Streptococcus suis* infections

Constance Schultsz and Kees C. H. van der Ark ^1^

^1^ Department of Medical Microbiology and Department of Global Health-Amsterdam Institute for Global Health and Development, Academic Medical Center, University of Amsterdam, The Netherlands

*Streptococcus suis* is an emerging zoonotic pathogen. Although many countries perform systematic surveillance of vaccine-preventable and other common causes of meningitis, *S. suis* meningitis is not a notifiable disease, with the exception of northern Thailand between 2008 and 2012. The burden of human *S. suis* infections is likely to be underestimated as indicated by case reports from an increasing number of countries [2,104,105,106,107,108,109], and human *S. suis* infections have now been reported from all continents. A recent study estimated the burden of disease caused by *S. suis* in Vietnam and reported annual disease incidence ranging from 0.249 to 0.324 per 100,000 population, in the years 2011–2014 [110]. Risk factors for human infection include consumption of raw pork blood or products; direct exposure to pigs, particularly in the presence of skin lesions; pig related occupations; and male sex [19,86,87]. Human-to-human transmission has not been demonstrated to occur to date.

*S. suis* serotype 2 is the main serotype causing zoonotic infections. Serotype 14 is known to also contribute significantly [111]. Other serotypes have been reported in single cases, including serotypes 1, 5, 9, 16, 21, and 31. The serotype is determined by the antigenic properties of the CPS of *S. suis*, which is encoded by multiple genes located in a single locus. The *cps* gene locus can be exchanged between *S. suis* of different serotypes, and in a study from The Netherlands, it was shown that such *cps* switch may lead to an increase in zoonotic potential. Zoonotic *S. suis* serotype 2 strains of ST20, isolated from human patients, were shown to be genetically highly related to serotype 9 strains of ST16. *S. suis* ST16 isolates have never been isolated from human patients but are highly implicated in invasive disease in pigs in The Netherlands [26]. The serotype 2 CPS appears to induce very little to no protective immunity, as was illustrated by a case of reinfection with *S. suis* serotype 2 in a Dutch slaughterhouse worker [112].

In addition to *cps* locus acquisition, loss or acquisition of other genes or loci may contribute to changes in zoonotic potential. For example, the *S. suis* ST20 strains acquired not only the serotype 2 *cps* locus but also a prophage carrying a type 1 Restriction-Modification (R-M) system (SsuCC20p) [113]. Such R-M systems have previously been shown to regulate gene expression through changes in methylation, driven by the phase variation of the specificity unit of the system [114]. Further analysis of this system in our lab indicates that it is indeed phase variable and that this variation results in differences in colony morphology. This observation suggests that *S. suis* ST20 strains may have acquired the ability to adapt to specific niches through the acquisition of a R-M system that can regulate CPS and/or other surface exposed molecules gene expression (manuscript submitted).

Ingestion of contaminated food items is an important route of infection with *S. suis*, especially in Southeast Asia. We have previously shown that the CPS modulates the interaction of *S. suis* with intestinal epithelial cells in vitro. Using human Caco-2 cells and porcine IPEC cells, we observed that serotype 2 strains adhered better to the human cells than serotype 9 strains, whilst the opposite was observed for the porcine cells to which serotype 9 strains adhered better. In addition, non-encapsulated mutant strains showed increased adherence, invasion, and translocation across Caco-2 cells, clearly indicating a role for the CPS in intestinal infection in this experimental model [54]. To further study *S. suis* intestinal translocation, models are needed that represent the intestinal tract better than colon carcinoma cells in continuous culture. Recently, developments in stem cell research have made it possible to grow intestinal organoids, which contain most cell types present in the proximal and distal intestinal mucosa ex vivo. In addition, these organoids can now be grown in two-dimensional structures, thus allowing study of translocation from lumen into mucosa, replicating intestinal translocation. Initial experiments with *Listeria monocytogenes*, another Gram-positive pathogen known to cause meningitis after intestinal translocation to the blood, demonstrated invasion and translocation patterns similar to those observed in mouse models and mouse organoids. The zoonotic *S. suis* serotype 2 strain BM407 was shown to translocate in this model as well, which makes it a promising model for future study of intestinal infection with *S. suis*.

*S. suis* is likely to continue to emerge as a zoonotic pathogen, given the pressure to reduce antimicrobial use in livestock because of the global threat of antimicrobial resistance. In addition, a universally effective vaccine that can be used in pigs is not available. The global export of pigs that carry multiple serotypes of *S. suis* will facilitate exchange of genetic material through recombination and mobile genetic elements that may result in selective advantage and niche adaptation. It is crucial to understand the factors that are involved in the ability to cause zoonotic infection and to monitor if this may eventually result in *S. suis* strains that are human adapted and have become human-to-human transmissible.

## Appendix F

**Proceeding from the 4th International Workshop on *Streptococcus suis***

The role of concurrent infections in predisposing to *Streptococcus suis* and other swine diseases

Susan L. Brockmeier ^1^

^1^ USDA, ARS, National Animal Disease Center, Ames, IA

Although we know that pathogens such as *Streptococcus suis* can be primary pathogens, causing disease on their own, experimental and anecdotal evidence from the field indicate that the incidence and severity of disease with certain pathogens like *S. suis* can increase when concurrent infections with additional pathogens occur. The last National Animal Health Monitoring Survey conducted by the Animal and Plant Health Inspection Service of the United States Department of Agriculture indicated that respiratory disease is the primary cause of both nursery and grower/finisher deaths in swine and that meningitis is the second most common cause of nursery deaths, which is primarily caused by *S. suis* and *Glaesserella* (*Haemophilus*) *parasuis*. The top ten reported primary etiologies of porcine respiratory disease cases at the Iowa State University diagnostic laboratory from 2013 to 2014 were PRRSV, SwIV, *Mycoplasma hyopneumoniae*, *Pasteurella multocida*, *S. suis*, unspecified bacteria, *Actinobacillus suis*, *G. parasuis*, *Actinobacillus pleuropneumoniae*, and *Bordetella bronchiseptica*, in that order. From the 3330 cases where SwIV was identified as the primary etiology, 75% had secondary bacterial pneumonia as well, with *S. suis*, *P. multocida*, *G. parasuis*, *B. bronchiseptica*, and *Escherichia coli* being the most prevalent bacteria isolated (Courtesy of Dr. Phillip Gauger, Iowa State University). These results were similar for cases where PRRSV was determined to be the primary aetiology. The term porcine respiratory disease complex has been used to describe this common multifactorial respiratory disease in swine that occurs as a result of polymicrobial infections, environmental stressors, and host factors such age and immunological status.

Coinfections can be the result of multiple viruses, bacteria, or a combination of viruses and bacteria infecting the pig at the same time or in close succession. Numerous examples of coinfections with common swine viruses such as PRRSV, SwIV, porcine circovirus (PCV), and PRCV have been detected in the diagnostic lab and investigated experimentally, and coinfections with these viruses generally result in a more severe clinical disease outcome [115,116,117,118]. Similarly, coinfections with the viruses mentioned above and common swine bacterial pathogens have often shown that these viruses are potent initiators of secondary bacterial infections [34,35,36,37,38,39,40,41,42]. Primary bacterial pathogens can also predispose to secondary bacterial colonisation and disease. Common examples of this in swine are enzootic pneumonia, where *M. hyopneumoniae* predisposes to secondary pneumonia with *P. multocida*, and atrophic rhinitis, where *B. bronchiseptica* predisposes to secondary infection with toxigenic strains of *P. multocida* [119,120,121]. Our lab has also demonstrated that *B. bronchiseptica* can increase colonisation with other bacteria such as *G. parasuis* [122]. Coinfections are not limited to only two pathogens and can be complicated in nature. For example, we have shown that PRRSV alone predisposes pigs to pneumonia with *B. bronchiseptica* but not with *P. multocida* whereas *B. bronchiseptica* can predispose to nasal colonisation with *P. multocida* [34]. However, if the pig is infected with both PRRSV and *B. bronchiseptica*, this can lead to pneumonia with *P. multocida* as well [123].

There are several mechanisms that contribute to increased susceptibility to secondary infection and the enhanced disease that often occurs with coinfections, including disruption of the epithelial barrier or alteration of the innate or adaptive immune responses by the primary pathogen, direct interactions between or among the pathogens such as the formation of multispecies biofilms, disturbances to the ecological niche such as the upper respiratory microbiota, or enhanced transmission. An example of mechanical disruption would be the epithelial damage caused by pathogens such as SwIV or *B. bronchiseptica* resulting in decreased cilial clearance and potentially in exposure of new attachment sites and systemic invasion [124,125]. Decreased clearance of bacteria by professional phagocytes such as alveolar macrophages and neutrophils can lead to secondary bacterial infections. Viruses such as PRRSV and SwIV and bacteria such as *B. bronchiseptica* have been shown to affect phagocyte clearance though such mechanisms as being directly cytotoxic, altering functions such as phagocytic or bactericidal ability, or inhibition of recruitment of phagocytes to the site of infection [126,127,128,129,130,131,132]. These actions can be due to direct interactions of the pathogens with the phagocytes or via altered cytokine production. Often, there is an altered cytokine response to coinfections compared to the response seen with a single pathogen resulting from signalling through multiple pattern recognition receptors that recognize pathogen-associated molecular patterns. Frequently, this results in an amplified inflammatory response with coinfections that lead to more severe clinical signs and enhanced lesions. We and others have shown that coinfection with PRRSV, SwIV, or PRCV and bacteria such as *B. bronchiseptica*, *S. suis*, or *G. parasuis* result in a greater incidence of disease, greater percentage of lung affected, more severe lesions, and slower resolution of lesions than occuring with infection with the single pathogen alone, and this is often correlated with an amplified pro-inflammatory cytokine response [35,39,43,44,45] (Brockmeier, unpublished data).

Direct interactions among pathogens can lead to enhanced infection with secondary pathogens as well. For example, it has been shown that SwIV can promote adherence, colonisation, and invasion of *S. suis* due to interaction with the capsular sialic acid moiety present on some serotypes [52,53,133]. It has also been demonstrated that certain species of bacteria such as *Bordetella* can enhance colonisation of other bacterial species by a process called piracy of adhesins, whereby one bacterial species can utilize adhesins secreted by another bacterial species to attach to host cells [134].

There has been a great deal of interest lately in examining the microbiome of the digestive and respiratory tract and in determining if and how the local community of microbes might influence gut and respiratory tract health and/or predisposition to infection. Studies currently or recently performed have shown that infection with pathogens such as SwIV, PRRSV, and *B. bronchiseptica* results in significant changes to the nasal microbial composition and significant increases in abundance of certain genera, such as *Actinobacillus* and *Streptococcus* (Brockmeier, unpublished data). In addition, antimicrobial usage typically used in swine production has been shown to result in similar changes to the microbiota [135]. Further studies will be needed in order to determine whether these changes in composition result in increased incidence of disease with pathogens such as *S. suis*.

Development of animal models that recapitulate the polymicrobial infections typically seen in swine production facilities can be challenging but are needed in order determine the best intervention strategies to prevent the clinical disease seen with coinfections. Many factors such as the virulence of the pathogens, immune status, age of the pig, order and timing of exposure to the pathogens, and possibly microbiome can all affect the success of developing a model that reproduces the polymicrobial diseases that are seen on the farm. Once a model is developed, intervention strategies such as vaccination, antimicrobials, or other biotherapeutics can be tested for their effectiveness at preventing or treating disease outbreaks. For instance, with coinfection models that we have developed, we have demonstrated that, for certain strains of PRRSV, vaccination can reduce the incidence of secondary bacterial pneumonia [136]. Others have shown that a previous low dose challenge with *S. suis* used to vaccinate pigs, but not previous PRRSV vaccination, resulted in a lower incidence of streptococcal disease in a PRRSV/*S. suis* coinfection model [137].

In conclusion, the intensification of animal agriculture has resulted in the circulation of numerous potential pathogens at any given time on swine farms, and coinfections are more likely the norm rather than the exception. This can result in increased incidence and enhanced disease with certain pathogens including *S. suis*. We are just beginning to determine the pathogenesis of these polymicrobial infections and to develop suitable models for their study. However, continued study in these areas is needed to be able to develop effective intervention strategies to prevent the effects of these diseases on swine production.

## Appendix G

**Proceeding from the 4th International Workshop on *Streptococcus suis***

Viral coinfection replaces effects of suilysin on adherence and invasion of *Streptococcus suis* into respiratory epithelial cells grown under air–liquid interface conditions

Peter Valentin-Weigand ^1^, Fandan Meng ^2,3^, Jie Tong ^2^, Désirée Vötsch ^1^, Ju-Yi Peng ^3^, Xuehui Cai ^2^, Maren Willenborg ^1^, Georg Herrler ^3^, and Naihuei Wu ^3^

^1^ Institute for Microbiology, University of Veterinary Medicine Hannover, Hannover, Germany
^2^ State Key Laboratory of Veterinary Biotechnology, Harbin Veterinary Research Institute, Chinese Academy of Agricultural Sciences, Harbin, China
^3^ Institute for Virology, University of Veterinary Medicine Hannover, Hannover, Germany

Coinfections with SwIV may affect the course of *S. suis* infections, as has been shown recently in a precision-cut lung slice model and in immortalized cells [52,53]. However, the molecular mechanisms underlying these coinfections are poorly understood. So far, it has been shown that the interaction of SwIV hemagglutinins with capsular sialic acids of *S. suis* results in the binding of streptococci to virus-infected cells [52,53]. This adherence mechanism seems to be even more efficient than binding mediated by bacterial adhesins [52]. The enhanced adherence and colonisation efficiencies of *S. suis* observed after coinfection with SwIV affect also the subsequent tissue invasion. Furthermore, a coinfection with SwIV provides an additional advantage because virus infection induces apoptosis of virus-infected cells, which are mainly ciliated cells. It has been proposed previously that the loss of these specialized cells enables bacterial pathogens to get access to subepithelial cells and, thus, to spread to other parts of the host organism [138,139].

So far, the mechanisms that enable *S. suis* to invade mucosal surfaces are not well known. A streptococcal protein that contributes to bacteria–host cell association is suilysin, though it is not a typical adhesin as it is not cell-bound but secreted [48]. This cholesterol-dependent cytotoxin can induce apoptosis, resulting in epithelial damage. In addition, it is able to mediate attachment of *S. suis* to the surface of the respiratory epithelium [48,49]. It has been suggested that suilysin facilitates the colonisation and the establishment of the initial stages of infection in pigs by promoting host cell lysis and invasion of *S. suis* through the epithelium when bacteria have colonised the upper respiratory tract [49,140]. Furthermore, *S. suis* lacking the suilysin-gene has been shown to be avirulent in a mouse infection model when applied via the intraperitoneal route but virulent in pigs after intravenous infection, which indicates that suilysin is not essential for *S. suis* to cause systemic infection in pigs once the bacteria have entered the blood vessels [141]. Nevertheless, suilysin-negative clinical isolates are also found frequently in swine populations worldwide [142,143]. The prevalent ratios of suilysin-positive and suilysin-negative strains worldwide are varying, and not all virulent strains of *S. suis* produce suilysin [142,143]. Therefore, analyzing the biological role of suilysin in different stages of bacterial infection may help to better understand the pathogenesis of non-cytotoxic *S. suis* strains.

In a recent study, we applied highly differentiated primary porcine respiratory epithelial cells grown under air–liquid interface conditions to analyse the contribution of SwIV to the virulence of *S. suis* with a special focus on its cytolytic toxin suilysin [51]. We found that, during secondary bacterial infection, suilysin of *S. suis* contributes to the damage of well-differentiated respiratory epithelial cells in the early stage of infection, whereas cytotoxic effects induced by SwIV became prominent at later stages of infection [51]. Prior infection by SwIV enhanced the adherence to and colonisation of porcine airway epithelial cells by the wildtype *S. suis* strain and a suilysin-negative *S. suis* mutant in a sialic-acid dependent manner. A striking difference was observed with respect to bacterial invasion. After bacterial mono-infection, only wild-type *S. suis* showed an invasive phenotype whereas the mutant remained adherent. When the epithelial cells were preinfected with SwIV, also the suilysin-negative mutant showed invasion capacity [51]. As mentioned above, suilysin is known to be a virulence-associated factor that facilitates *S. suis* invasion as shown for both immortalized cells and differentiated airway epithelial cells [48,49]. The cytolytic activity of suilysin contributes significantly to this effect, as shown in our previous study, in which a mutant expressing a point-mutated suilysin lacking cytolytic activity was significantly less invasive than the wild-type streptococci [49]. Nevertheless, there was a significant difference between the invasion rates of two mutants lacking only the cytotoxic activity or lacking the whole protein [49]. This finding suggests that there may be invasion-promoting effects of suilysin which do not depend on the cytolytic activity.

Taken together, suilysin-negative *S. suis* strains can become invasive in a coinfection scenario with SwIV, which might explain why suilysin-negative strains can cause clinical infections. Finally, these results may contribute to our understanding of viral–bacterial interactions on airway coinfections.

## Appendix H

**Proceeding from the 4th International Workshop on *Streptococcus suis***

Switching of capsular type impacts on *Streptococcus suis* virulence in mice and pigs

Masatoshi Okura ^1^, Jean-Philippe Auger ^2^, Tomoyuki Shibahara ^3,4^, Guillaume Goyette-Desjardins ^2^, Marie-Rose Van Calsteren ^5^, Fumito Maruyama ^6,7^, Makoto Osaki ^1^, Mariela Segura ^2^, Marcelo Gottschalk ^2^, and Daisuke Takamatsu ^1,8^

^1^ Division of Bacterial and Parasitic Diseases, National Institute of Animal Health, National Agriculture and Food Research Organization, Tsukuba, Japan
^2^ Faculty of Veterinary Medicine, University of Montreal, Saint-Hyacinthe, Quebec, Canada
^3^ Division of Pathology and Pathophysiology, National Institute of Animal Health, National Agriculture and Food Research Organization, Tsukuba, Japan
^4^ Department of Veterinary Science, Graduate School of Life and Environmental Sciences, Osaka Prefecture University
^5^ Saint-Hyacinthe Research and Development Centre, Agriculture and Agri-Food, Saint-Hyacinthe, Quebec, Canada
^6^ Microbial Genomics and Ecology, Office of Industry-Academia-Government and Community Collaboration, Hiroshima University
^7^ Scientific and Technological Bioresource Nucleus, Universidad de La Frontera, Temuco, Chile
^8^ The United Graduate School of Veterinary Sciences, Gifu University, Gifu, Japan

*Streptococcus suis* is an important zoonotic pathogen causing various diseases in pigs and humans [1]. Strains of *S. suis* can be classified into different serotypes on the basis of antigenic differences in CPS [1,2]. Among more than 30 serotypes, serotype 2 is the most frequently associated with clinical cases in both pigs and humans [2]. Meanwhile, CPS of *S. suis* is known to be a major virulence factor contributing to protection against phagocytosis by host cells [3]. In addition, several studies demonstrated attenuated virulence of the isogenic non-encapsulated mutants by in vivo murine and porcine models [3]. However, almost all of the studies used serotype 2 strains, and little is known on the roles of CPSs of other serotypes as a virulence factor. Therefore, it is unknown whether differences in serotype themselves (i.e., differences in CPS structure) directly affect *S. suis* virulence. To answer this question, we experimentally generated six serotype-switched mutants using the serotype 2 strain P1/7 (P1/7cps2to3, P1/7cps2to4, P1/7cps2to7, P1/7cps2to8, P1/7cps2to9, and P1/7cps2to14) by exchanging the CPS synthesis gene cluster for those of serotypes 3, 4, 7, 8, 9, and 14, respectively, and compared their virulence in mice and pigs. In mice, *S. suis* strain/mutants were intraperitoneally inoculated at a final dose of 1 × 10^7^ CFU to groups of 10–11 six-week-old C57BL/6 mice for survival (14 days postinfection) and blood bacterial burden (24, 48, and 72 h postinfection) evaluation. P1/7cps2to8, expressing serotype 8 CPS, showed higher mortality and blood bacterial load than serotype 2 strain P1/7, with all infected mice succumbing within 24 h from septic shock. The virulence of P1/7cps2to7, P1/7cps2to9, and P1/7cps2to14 were equivalent to that of P1/7 (survival: approximately 40% of infected animals), whereas the mortality of mice inoculated with P1/7cps2to3 and P1/7cps2to4 was remarkably reduced compared with that of P1/7, with 70% and 100% of survived animals, respectively. In pigs, one hour after intranasal administration of 1% acetic acid, groups of 4–5 crossbreed specific pathogen-free piglets aged 5 weeks were intranasally inoculated with approximately 2 × 10^9^ CFU of respective *S. suis* strain/mutants to monitor for clinical signs for 7 days postinoculation and to evaluate bacterial recovery from infected animals. Clinical signs such as lameness, shivering, and dysstasia were observed only with P1/7, P1/7cps2to7, and P1/7cps2to8 strains. Inoculated strains were recovered from several major organs and blood from the pigs showing clinical signs infected with P1/7 and P1/7cps2to8, while P1/7cps2to7 was recovered only from brain. In addition, P1/7cps2to8 was also recovered from several major organs of experimentally infected but clinically healthy pigs. Except for one of the pigs infected with P1/7cps2to14, in which the inoculum was recovered from several major organs and blood, recovery of infected bacteria from blood or more than two of major organs was not observed in infected pigs that showed no clinical signs during the experiment period. Taken together, our data indicate that serotype switch in *S. suis* has different impacts on the virulence in mice and pigs depending on switched serotypes. This suggests that CPS structure itself is an important factor in determining levels of *S. suis* virulence, although further studies are needed to elucidate the mechanisms behind the observed phenomena.

## Appendix I

**Proceeding from the 4th International Workshop on *Streptococcus suis***

Treatments for *Streptococcus suis* in the light of evolution

Lucy A. Weinert ^1^

^1^ Department of Veterinary Medicine, University of Cambridge, Cambridge, UK

Bacterial pathogens can and do evolve against treatments. The most obvious of such change is the acquisition of resistance to antimicrobials. Even by the early 1980s, large numbers of *S. suis* isolates from pigs had become resistant to tetracycline and macrolide classes of antibiotics [144], and more recently, some isolates have become resistant to all classes of antibiotics used in pigs [145,146]. Resistance also arises against vaccines [147]. *S. suis* bacterin vaccines consist of strains isolated from a herd, which are then inactivated and administered as treatment. There has been no consistent evidence that bacterin vaccines give cross-protection against different strains of the bacterium, and so vaccinated animals can develop *S. suis* clinical disease [148]. The failure of these *S. suis* vaccines is therefore different from acquired resistance. However, treating only a subpopulation of *S. suis* could lead to vaccination-driven strain replacement or to vaccination-driven bacterial evolutionary change (acquired vaccine resistance). These processes will decrease vaccine effectiveness over time. Given our knowledge of the ecology and genetics of *S. suis*, it is important to consider how *S. suis* might respond and evolve against new and existing treatments and whether we can use this information to design treatments that are more “evolution-proof”.

The lack of cross-protection of bacterin vaccines speaks to the considerable genetic diversity of *S. suis*, which makes it a challenge to design a universal vaccine. A universal vaccine would need to contain an antigen that is highly conserved at the protein level across the whole *S. suis* species. Unfortunately, less than half the proteins found in an average *S. suis* strain are conserved across the species at more than 80% protein identity [64]. This is without considering that lineages of “diverse *S. suis*”, which cause typical *S. suis* disease, are found colonising the same niches and freely recombine with *S. suis* [84]. Their limited conservation with “normal *S. suis*” at the amino acid level substantially decreases the number of species-wide conserved proteins [84]. It is doubtful that a suitable universal antigen exists, but if it did and was able to induce mucosal immunity (i.e., immunity against carriage of *S. suis*), we could eliminate *S. suis* from pig populations and deliver herd immunity effects, protecting pigs that had not been vaccinated. *S. suis* is, however, an extremely common commensal, found in the tonsils of most and perhaps all pigs. Wiping out a ubiquitous part of the microbiome may have unforeseen effects on other common opportunistic pathogens inhabiting the upper respiratory tract of pigs, such as *Haemophilus parasuis* and *Actinobacillus pleuropneumoniae*.

Another more feasible strategy would be to selectively target strains that are more prone to causing clinical disease. Our lab and others have shown many trends in genetic and phenotypic differences between strains isolated from the upper respiratory tract of pigs without *S. suis* clinical signs (hereafter “nonclinical”) and strains isolated from the lungs or systemic sites of pigs with *S. suis* clinical signs (hereafter “clinical”) (Figure A1) [64,145,146,149]. Overall, the genetic diversity of clinical *S. suis* is less than that of nonclinical strains, implying that we need not design a universal vaccine to eliminate the majority of *S. suis* disease.

One obvious candidate for a vaccine targeting clinical strains is the CPS outer layer of *S. suis*. This is because the majority of clinical strains have a capsule (Figure A1), which helps them to survive in the blood [150]. Indeed, polysaccharide capsules are widely used or considered in treatments for other streptococcal pathogens. However, capsules evolve rapidly. Because they are genetically diverse, multiple different polysaccharides are needed in a vaccine to target different strains. As with bacterin *S. suis* vaccines, there will be vaccine failure if we do not target all CPS types. Not only that, we may get vaccination-driven strain replacement of the population, and this can reduce the overall vaccine effectiveness over time as non-vaccine types rise in frequency. This has been suggested as a reason for the rise in non-vaccine strains in invasive pneumococcal disease [151]. In addition to their diversity, capsule loci are often recombination “hot-spots”, and this gives rise to frequent and easy capsule “switching” between strains [152]. This means that highly invasive strains can quickly evolve non-vaccine CPS types, leading to acquired vaccine resistance. For these reasons, it would be desirable to identify alternative virulence candidates. Proteins with higher conservation and in regions of the genome with greater syntenic preservation and less recombination would be obvious choices.

There may also be inherent problems with targeting only clinical strains. Despite the worldwide use of beta-lactams in pigs for over 50 years, the majority of clinical *S. suis* remain sensitive to these antibiotics. However, beta-lactam resistant strains do exist and are primarily found in commensal sites (Figure A1). These resistant strains could spread if the susceptible (clinical) strains were wiped out.

The problems discussed above—strain replacement/evolution—will be reduced if we exclusively induce systemic immunity (i.e., immunity against systemic infection that has no effect on colonisation). This is because *S. suis* is an opportunistic pathogen and so commensal sites (and not clinical sites) are the site of transmission of *S. suis* to other pigs. If the *S. suis* flora in commensal sites remain unaffected by vaccination, transmission dynamics remain unchanged. We would still get vaccine failure for any strains that were not targeted by the vaccine, but the effectiveness of this vaccine will not decrease over time. Unfortunately, systemic immunity may be less successful in protecting against *S. suis* disease than mucosal immunity. For example, pneumococcal vaccines triggering systemic immunity with protection against invasive disease appear to be less effective against pneumonia [153].

To summarise, existing data on *S. suis* allow us to draw some important conclusions. First, if we do not have a universal target against all *S. suis*, we cannot rule out strain replacement/evolution, leading to decreased vaccine effectiveness over time, with potential downstream effects on antimicrobial effectiveness. Second, and more positively, *S. suis* strain replacement/evolution should not be a problem with vaccines that induce systemic but not mucosal immunity. These are considerations that we can bear in mind in designing new vaccines but are minor in view of the many other existing challenges in bringing an effective *S. suis* vaccine to market.

## Appendix J

**Proceeding from the 4th International Workshop on *Streptococcus suis***

Perinatal antimicrobials: friend or foe?

Virginia Aragon ^1^

^1^ IRTA, Centre de Recerca en Sanitat Animal (CReSA, IRTA-UAB), Campus de la Universitat Autònoma de Barcelona, Bellaterra, Spain

*Streptococcus suis* is an early coloniser of the upper respiratory tract of piglets, which are commonly colonised by *S. suis* from birth. In fact, piglets can get colonised as soon as they pass through the birth canal, since *S. suis* is found in the sow vagina. Virulence of the *S. suis* strains and the immunity of the animals influence the outcome of the infection. *S. suis* infections are one of the main causes of antimicrobial usage in piglets. Due to the young age of the piglets affected and the lack of an effective commercial vaccine, many farms use metaphylactic perinatal antimicrobials to control of *S. suis* disease. A similar situation is found with the diseases produced by other early colonisers of piglets, such as *Haemophilus parasuis* and *Mycoplasma hyorhinis*. Nowadays, public and private institutions are urging for a significant reduction in antimicrobial use, since the emergence of drug resistances has become a major health problem. An additional problem of antimicrobial usage is that these treatments can also affect the beneficial bacteria of the microbiota.

Microbiota is the community of microorganisms that live on different tissues on all multicellular organisms. Pathogen exclusion is an important role of the microbiota [154], and it can be achieved by competition or by induction of the correct maturation of the immune system. Thus, the microbiota is essential for health, and this has changed our current view on antimicrobials, since they can have deleterious effects on favourable bacterial populations [155]. However, the use of metaphylactic antimicrobials at early stages of life is still in practice in porcine production.

Clinical observations indicate that overuse of antimicrobials at an early age can have a harmful effect later in the pigs’ health [156]. In an attempt to understand this phenomenon, the outcome of perinatal antimicrobial treatment on the nasal microbiota at weaning was studied in two farms [157]. These two farms reported polyserositis cases in the nursery phase and used antimicrobials during the lactation phase for disease control. The nasal microbiota was determined when antimicrobial treatment was used early in life and later when antimicrobial treatments were eliminated during the lactation period. Elimination of perinatal antimicrobials resulted in an increase in bacterial diversity in the nasal microbiota at weaning. The specific analysis of the relative abundance of *Streptococcus* showed a limited reduction of this genus at weaning after removal of antimicrobials in both farms. However, interpretation of this information is not straightforward since the genus *Streptococcus* includes several species, some of which cannot be considered pathogenic. Furthermore, *S. suis* strains display a wide variety of virulence capacity, complicating the interpretation of the result on streptococcal abundance with respect to health consequences. Further analysis of the microbiota composition found that the elimination of antimicrobials produced an increase in the relative abundance of *Prevotella* and *Lactobacillus* and a decrease in *Moraxella* and *Bergeyella* in both farms. While *Prevotella* and *Lactobacillus* are considered important components of a healthy microbiota, *Moraxella* and *Bergeyella* have been reported to have pathogenic potential. In agreement, these changes in microbiota composition were accompanied by an improvement of the piglets’ health and a higher productivity in the nursery phase. Mortality (in one of the farms) and medication cost in the nursery (in both farms) were reduced, indicating that management of lactation without perinatal antimicrobials had a beneficial impact later in life. Thus, the changes observed after elimination of antimicrobials can be explained by the consequent reduction of pathogenic genera and the simultaneous increase of beneficial bacterial genera, together with the general increase in microbiota diversity, which has been widely associated with improved health status.

A different study showed that perinatal ceftiofur administration delayed the colonisation by *S. suis* and the pathogen load. However, a detailed analysis of the *S. suis* strains showed that different strains were selected by the antimicrobial drug and that serovars associated with virulence were more abundant in the nasal cavities of the treated piglets (unpublished results). While these results need deeper analysis, they indicate that the precise effects of antimicrobials are difficult to predict.

Early medication with antimicrobials has to be carefully considered since antimicrobials interfere with the establishment of the microbiota and, in consequence, with immune maturation and other microbiota functions, which may have a lasting health effect later in life.

## Appendix K

**Proceeding from the 4th International Workshop on *Streptococcus suis***

Manufacture, control, and use of autogenous vaccines within European Union—the regulatory framework

Mariette Saléry ^1^

^1^ French agency for veterinary medicinal products - French agency for food, environmental and occupational health safety (Anses-ANMV), France

Controlling *S. suis* in the field may require the use of autogenous vaccines since no other vaccines are nowadays licensed in Europe. Obviously, the wide use of autogenous vaccines and cross-border movement of animals vaccinated with autogenous vaccines is now a common practice within Europe. Therefore, harmonising quality of these products has been considered necessary by the Coordination Group for Mutual Recognition and Decentralised Procedure—Veterinary (CMDv) and the national competent authorities for Veterinary Medicinal Products (VMPs) in Europe.

Definitions:

The current European directive 2001/82 [158] that regulates VMPs defines autogenous vaccines but does not provide any other requirements. Article 3(1) states that the directive shall not apply for “Inactivated immunological VMPs which are manufactured from pathogens and antigens obtained from an animal or animals from a holding and used for the treatment of that animal or the animals of that holding in the same locality”. Article 4, about non-inactivated autogenous vaccines, says that the Member States (MS) may provide that the directive shall not apply. In other words, the inactivated autogenous vaccines fall under national legislation whereas live autogenous vaccines may be under European regimen (so should be authorized as any other licensed vaccines) or national regimen according to the MS decision.

In January 2019 a new legislation on VMPs has been adopted in the European Union, the regulation 2019/6 [159], applicable in January 2022. In this new regulation, autogenous vaccines have an updated definition and fall under European regimen. Article 2(3) of this regulation requires that some articles of the regulation apply to “inactivated VMP manufactured from pathogens and antigens obtained from an animal or animals in an epidemiological unit and used for the treatment of that animal or those animals in the same epidemiological unit or for the treatment of an animal or animals in a unit having a confirmed epidemiological link”.

Background:

In 2015, the CMDv started a work on autogenous vaccines and overviewed the different situations and regulations in Europe [160]. The outcome was that European MS had various experiences and backgrounds with respect to the autogenous vaccines manufacture and use. As an example, in France, where only bacterial autogenous vaccines are allowed, the manufacturer should be authorized to prepare the autogenous vaccine before marketing the autogenous vaccine.

The French agency authorizes the preparation based on four pillars:

- The preparation site (called establishment), authorized based on inspection and a technical dossier
- The qualified person, mentioned in the authorization as the person who prepares and releases the product
- A positive list of bacteria and animal species, in annex of the license given to the manufacturer and based on the lists of disease for which no authorized vaccines with marketing authorization is available as an alternative. In cases when autogenous vaccine is needed when a vaccine is already authorized but in lack of efficacy situation, the manufacturer should seek a derogation.
- A positive list of adjuvants, based on the adjuvants used by the manufacturer and in compliance with the Maximum Residues Limit regulation.

The French market is displayed in Figure A2.

Recommendations paper and new veterinary regulation:

Then, the CMDv has prepared a recommendation paper [78] which deals with the manufacture, the control, and the use of viral and bacterial autogenous vaccines in Europe. In the framework of the new regulation, this paper will be updated in the coming years. The same locality has been defined as the same and single rearing site, farm where the pathogen is present or multiple sites having an epidemiological link. It has been clarified that groups of animals have an epidemiological link when one of them is to be put in contact with pathogens it has never met before but which are present in other group of animals raised in another rearing site or farm. The movement between sites should be considered. It reflects the actual situation in Europe and current field practices in integrated systems for poultry, fishes, and pigs especially. The introduction of the concept of epidemiological link allows now the use of autogenous vaccines in parental lines or, for animals, prior introduction in fattening sites where they will be in contact with new pathogens. In the new regulation, the concept is now directly in the definition, article 2(3).

The paper states the following:

- Autogenous vaccines should be used only to solve an exceptional epidemiological situation, not in replacement of the regular authorized VMP. It should be shown that no licensed immunological VMP is available under the cascade prescriptions or that licensed immunological VMP have been proved to be not efficacious for the specific situation.
- Specific pathogen should have been isolated in the locality, during an outbreak of the disease.
- The veterinarian should have made the diagnosis and is responsible of the administration. They are also obliged to report any quality defects and adverse events as regulated by the provisions for the Pharmacovigilance.
- Manufacture should be done under Good Manufacturing Practices (GMP) or GMP-like requirements. The manufacturers should have been authorized, and the compliance with the requirements is controlled by inspection. A qualified person should be designated to ensure the quality of each batch. Transmissible Spongiform Encephalopathies’ (TSE) guidance should be followed for the starting materials.
- A veterinarian should make the diagnosis, collect the antigen, and ensure the traceability whereas an authorized competent site (a laboratory) should ensure the isolation and the identification.
- Starting materials should comply with the European Pharmacopoeia (Ph. Eur.) requirements; must be sterile; and, if of biological origin, must comply with TSE and extraneous agents requirements. Seed material should be pure, and inactivation should be validated.
- Batches should be controlled for sterility, complete inactivation, and endotoxin, if relevant.

All these disposals are partially now in articles 94, 105, 106(5), and 123 of the new regulation 2019/6, whereas 70 adds the provisions that manufacture should be in accordance with the principles of GMP and that guidelines for GMP should be developed for the autogenous vaccines.

Conclusion:

The autogenous vaccines in EU are a very interesting therapeutic tool to fight against diseases, especially *S. suis*. The current and future regulatory framework ensure high quality for those products. Safety and efficacy are not regulated. However, safety and efficacy of these products are better demonstrated through field use and laboratory research.
